# Thymol Derivatives as Antimalarial Agents: Synthesis, Activity Against *Plasmodium falciparum*, ADMET Profiling, and Molecular Docking Insights

**DOI:** 10.3390/biomedicines14010123

**Published:** 2026-01-08

**Authors:** Amatul Hamizah Ali, Rini Retnosari, Siti Nur Hidayah Jamil, Nur Aqilah Zahirah Norazmi, Nabel Darwish Zuhaidi, Su Datt Lam, Sylvia Chieng, Hani Kartini Agustar, Kuhan Chandru, Nurhezreen Md Iqbal, Lau Yee Ling, Jalifah Latip

**Affiliations:** 1 Department of Chemical Sciences, Faculty of Science and Technology, Universiti Kebangsaan Malaysia (UKM), Bangi 43600, Selangor, Malaysia; amatulhamizahali@yahoo.com.my (A.H.A.);; 2 Department of Chemistry, Faculty of Mathematics and Natural Sciences, State University of Malang (Universitas Negeri Malang), Jl. Semarang No. 5, Malang 65145, Indonesia; 3 International Joint Department of Materials Science and Engineering Between National University of Malaysia and Gifu University, Graduate School of Engineering, Gifu University, 1-1 Yanagido, Gifu 501-1193, Japan; 4 Department of Parasitology, Faculty of Medicine, Universiti Malaya, Kuala Lumpur 50603, Malaysia; 5 Department of Applied Physics, Faculty of Science and Technology, Universiti Kebangsaan Malaysia (UKM), Bangi 43600, Selangor, Malaysia; 6 Department of Biological Sciences and Biotechnology, Faculty of Science and Technology, Universiti Kebangsaan Malaysia (UKM), Bangi 43600, Selangor, Malaysia; 7 Department of Earth Science and Environment, Faculty of Science and Technology, Universiti Kebangsaan Malaysia (UKM), Bangi 43600, Selangor, Malaysia; 8 Space Science Center (ANGKASA), Institute of Climate Change, Level 3, Research Complex, Universiti Kebangsaan Malaysia (UKM), Bangi 43600, Selangor, Malaysia; 9 Malaysia Genome and Vaccine Institute, Jalan Bangi Lama, Kajang 43000, Selangor, Malaysia

**Keywords:** ADMET profiling, antimalarial agents, molecular docking, *Plasmodium falciparum*, thymol derivatives

## Abstract

**Background:** Thymol, a natural phenol with antimicrobial and antioxidant activities, and its derivatives offer promising scaffolds for antimalarial drug development, potentially helping overcome resistance. **Materials and Methods:** In this study, thymol derivatives were synthesized and assessed as antiplasmodial agents against both resistant and sensitive strains of *P. falciparum*, as well as *Plasmodium knowlesi*. The ligand molecules were assessed with *Plasmodium falciparum* chloroquine resistance transporter (PfCRT)’s potential using in silico molecular docking and ADMET analysis. The parent compound, thymol, was chemically modified through esterification and conjugation with hydroxybenzoic acid and cinnamic acid derivatives to generate analogs with varied substitution patterns. **Results:** The findings showed that among seven successfully synthesized thymol derivatives, compounds **4** and **6** exhibited notable potency against *Plasmodium falciparum* 3D7 (EC_50_ = 6.01 ± 1.7 µM and 6.8 ± 1.1 µM, respectively) with high SI values (16.5 and 14.6, respectively), indicating improved selectivity relative to thymol. The cytotoxicity evaluation against HCF mammalian cells revealed that most thymol derivatives were non-toxic, with CC_50_ values greater than 99 µM, except for compound 3 (CC_50_ = 71.4 ± 4.5 µM) and compound 1 (CC_50_ = 58.4 ± 2.3 µM), which exhibited moderate cytotoxic effects. The molecular docking results showed that compounds **3** (−8.4 kcal/mol), **4** (−8.3 kcal/mol), and **6** (−8.3 kcal/mol) exhibited strong binding affinities toward the PfCRT protein. **Conclusions:** Therefore, thymol derivative compounds **4** and **6** exhibited stronger antiplasmodial activity in vitro against *P. falciparum* and *P. knowlesi* with safety profiles against mammalian cells, targeting PfCRT, highlighting their potential as lead antimalarial candidates.

## 1. Introduction

Malaria is an infectious disease caused by the protozoa parasite of the genus Plasmodium like *P. falciparum* and *P. knowlesi*, which can be transmitted through the bite of the female *Anopheles* spp. mosquito. According to World Health Organization (WHO), the World Malaria Report estimates that in 2023 there were about 263 million malaria cases and 597,000 deaths worldwide, with Africa bearing the greatest burden, responsible for nearly 95% of all reported cases and 96% of global deaths [1]. South East Asian countries are known for the emergence of resistant cases. Drug resistance in *Plasmodium falciparum* remains a major challenge in malaria control, with multiple studies across Southeast Asia highlighting genetic markers associated with reduced drug efficacy. Artemisinin resistance, primarily linked to mutations in the kelch13 (K13) propeller domain such as C580Y, Y493H, and R539T, has been widely documented in Cambodia, Vietnam, Myanmar, and neighboring regions [2,3,4]. These mutations impair parasite clearance and have been associated with treatment failures in artemisinin-based combination therapies (ACTs). In addition, resistance to partner drugs such as piperaquine has been associated with plasmepsin 2–3 gene amplification and mutations in exonuclease E415G, further compromising the efficacy of dihydroartemisinin–piperaquine regimens [5,6]. Chloroquine resistance, which spread globally decades earlier, continues to be linked to mutations in the PfCRT transporter gene, including K76T and novel polymorphisms [6,7]. Similarly, mutations and copy number variations in PfMDR1 contribute to altered susceptibility to mefloquine and lumefantrine [8]. Collectively, these findings underscore the dynamic evolution of *P. falciparum* resistance, necessitating continued molecular surveillance and the development of novel therapeutic strategies to sustain malaria control efforts.

The chloroquine resistance transporter gene (CRT) represents one of the most pivotal genetic determinants of antimalarial resistance in *Plasmodium falciparum*. This gene encodes a transmembrane protein situated on the membrane of the parasite’s digestive vacuole, where it plays a crucial role in solute transport. Mutations in PfCRT, notably the K76T substitution, are strongly linked to chloroquine resistance by disrupting drug accumulation and promoting efflux from the vacuole [9,10]. Such alterations have severely undermined the clinical effectiveness of chloroquine (CQ), formerly the cornerstone of malaria treatment, leading to its withdrawal from global treatment protocols. The rapid dissemination of resistant *P. falciparum* strains emphasizes the urgent demand for innovative therapeutic strategies. Moreover, the continued emergence of resistance in *P. falciparum* reinforces the critical need for sustained drug discovery initiatives to develop novel antimalarial agents [11,12]. To address the growing challenge of antimalarial drug resistance, there is an urgent need to explore bioactive compounds derived from natural products as potential therapeutic agents, while also recognizing advances in medicinal chemistry as a relevant source of novel antimalarial agents [13,14].

Natural products continue to offer a rich source of antimalarial leads, particularly in the context of rising resistance to frontline therapies. Plant-derived terpenoids and phenolic compounds have been widely investigated for their antiplasmodial properties due to their structural diversity and ability to modulate parasite redox homeostasis [15]. Thymol, a natural phenolic monoterpenoid extracted from Thymus species and other plants, demonstrates significant antimalarial activity against *Plasmodium falciparum* [16,17]. Kumar et al. [17] reported that 4-chlorothymol inhibited growth of both CQ-sensitive (IC_50_ = 0.93 ± 0.24 μg/mL) and CQ-resistant strains (IC_50_ = 2.40 ± 0.42 μg/mL). Specifically, 4-chlorothymol, a thymol derivative, exhibits enhanced antiplasmodial activity by increasing reactive oxygen and nitrogen species levels and disrupting the parasite’s redox defense system through modulation of glutathione S-transferase (GST) and glutathione reductase (GR) enzyme activities [17]. Chemical modification of thymol markedly enhanced its antimalarial activity. Compared with thymol-based chalcones, all synthesized pyrazoline derivatives inhibited *P. falciparum* NF54 with IC_50_ values below 10 μM. Halogenated and nitro-substituted analogues showed the greatest potency, suggesting that increased lipophilicity and electron-modifying groups on the aromatic ring improve parasite sensitivity [18].

Beyond antimalarial properties, thymol exhibits broad therapeutic benefits including antimicrobial, antioxidant, anti-inflammatory, and antileishmanial activities [19,20], making it a promising scaffold for developing new antiparasitic agents. Dell’Agli et al. [21] evaluated thymol and *Satureja thymbra* oil and observed in vitro IC_50_ values of approximately 10–20 μg/mL against both CQ-sensitive and CQ-resistant Plasmodium strains, with a mode of action that likely involves plasmepsin II inhibition and interference with the first nuclear division. Fröhlich et al. [22] synthesized thymoquinone–artemisinin hybrids that inhibited the 3D7 strain at 3.7–5.9 nM, outperforming artesunic acid (8.2 nM). In contrast, thymol-based pyrazolines and chalcones were examined without accompanying quantitative details [23]. Similarly, Johnson-Ajinwo et al. [24] demonstrated that thymoquinone analogues bearing nitrogen, alkyl, or halogen substituents produced moderate antiplasmodial effects, suggesting the substituent type and position strongly influence activity. These hybrids, designed through ether linkages and esterification, exploit synergistic mechanisms of the parent scaffolds, highlighting the therapeutic potential of thymol-derived hybrids. Overall, SAR investigations suggest that halogenation, heterocyclic extension, and hybridization are the most promising modifications for thymol derivatives. These strategies improve pharmacological activity, metabolic stability, and strain coverage, underscoring the value of thymol scaffolds as templates for next-generation antimalarial agents.

In this study, the parent compound thymol was structurally modified via esterification to introduce a linker, followed by conjugation with hydroxybenzoic acid and cinnamic acid derivatives, yielding a series of derivatives with distinct substitution patterns. The resulting derivatives included hydroxybenzoate conjugates and cinnamate derivatives. These modifications introduced hydroxy (–OH) and halogen (Cl) groups, which are key determinants of physicochemical properties, biological activity, and pharmacokinetic profiles. All thymol derivatives were tested for their antiplasmodial activity through an in vitro drug susceptibility test against the zoonotic parasite *P. knowlesi* and non-zoonotic parasite *P. falciparum*, followed by molecular docking on PfCRT and ADMET analyses. The present study is essential to elucidate the role of specific functional moieties in thymol derivatives, particularly hydroxy and halogen groups, in enhancing antimalarial efficacy and potentially preventing the emergence of CQ resistance strains and the transmission of malaria.

## 2. Materials and Methods

### 2.1. Materials and Analytical Procedures

Commercial reagents were used without further purification. Anhydrous organic solvents were prepared using standard drying procedures, while other reagent-grade solvents were used as supplied. Reactions requiring dry conditions were carried out under an argon atmosphere. Analytical TLC was performed on Merck (Darmstadt, Germany) silica gel 60 F254 plates (No. 5715). Column chromatography employed Kanto silica gel 60N (neutral, spherical; particle sizes 40–50 or 63–210 μm). ^1^H (400 MHz) and ^13^C (100 MHz) NMR spectra were recorded on a Bruker Avance III spectrometer (Billerica, MA, USA) at the i-CRIM Laboratory, UKM, using acetone-d_6_ as the solvent and TMS as the internal standard. ^1^H NMR data are reported as chemical shift (δ), multiplicity, coupling constants (*J*, Hz), and integration. Multiplicities are indicated as s (singlet), br s (broad singlet), d (doublet), dd (doublet of doublets), t (triplet), q (quartet), quin (quintet), spt (septet), and m (multiplet).

### 2.2. Synthesis of Thymol Derivatives (***1***–***7***)

The synthesis of compounds **1**–**7** was carried out according to protocols reported in previous studies with slight modification [25,26]. Briefly, the procedure consisted of a two-step reaction sequence, which is outlined in the following section.

#### 2.2.1. Synthesis of 5-Isopropyl-2-methylphenyl 2-chloroacetate (**8**)

In the first step, compound **8** was synthesized as the key intermediate for compounds **1**–**7**. Triethylamine (5.0 mmol, 0.7 mL) and thymol (5.0 mmol, 0.75 g) in dichloromethane (25 mL) were cooled to 0 °C, and chloroacetyl chloride (5.0 mmol, 0.40 mL) in dichloromethane (2.6 mL) was added dropwise over 1 h under continuous stirring at 0 °C. The mixture was then stirred at room temperature for 3 h, washed with water, and dried over anhydrous MgSO_4_. After filtration and solvent removal under reduced pressure, the crude product was purified by silica gel column chromatography (n-hexane/ethyl acetate, 95:5, *v*/*v*) to afford compound **8** as a colorless oil (0.89, 4.0 mmol, 80%). ^1^H NMR (400 MHz, Acetone-*d*_6_) δ 1.27 (d, *J* = 8 Hz, 6H), 2.33 (s, 3H), 3.18 (m, 1H), 4.28 (s, 2H), 6.97 (s, 1H), 7.08 (dd, *J* = 4, 8 Hz, 1H), 7.31 (d, *J* = 8 Hz, 1H). The obtained NMR data for compound **8** were consistent with those reported in the literature [25] (Refer Supplementary Information).

#### 2.2.2. Synthesis of Compounds **1**–**7**

In the second step, compounds **1**–**7** were synthesized from intermediate **8**. Appropriate hydroxy-benzoic/cinnamic acid (2.5 mmol), compound **8** (2.5 mmol, 0.55 g), potassium iodide (2.5 mmol, 0.42 g), triethylamine (2.5 mmol, 0.35 mL) was stirred in dimethylformamide (25 mL) at rt for 24 h. The mixture was poured into ice, extracted with EtOAc (3 × 25 mL), concentrated, and purified by silica gel chromatography (n-hexane/EtOAc, 4:1) to give compounds **1**–**7**. Structural confirmation of all synthesized compounds was achieved by ^1^H and ^13^C NMR spectroscopy. For previously reported derivatives (**1**, **2**, and **4**–**6**), NMR spectra were re-recorded to verify structural identity and to maintain consistency across the dataset. The obtained spectra were consistent with those reported in the literature [25]. Compounds **3** and **7** represent newly synthesized derivatives, and their NMR data are reported here for the first time.

**2-[5-Methyl-2-(propan-2-yl)phenoxy]-2-oxoethyl 4-hydroxybenzoate (1)** white solid (0.64 g, 1.9 mmol, 78%), ^1^H NMR (400 MHz, Acetone-*d*_6_) δ 1.20 (d, *J* = 6.8 Hz, 6H), 2.27 (s, 3H), 3.16 (m, 1H), 5.18 (s, 2H), 6.93 (d, *J* = 2.0 Hz, 1H), 7.05 (m, 3H), 7.24 (d, *J* = 8.0 Hz, 1H), 7.18 (d, *J* = 8.0 Hz, 1H), 8.08 (m, 2H), 9.40 (s, OH). ^13^C NMR (100 MHz, Acetone-*d*_6_) δ 21.67, 24.26, 28.45, 62.54, 117.11, 122.13, 124.24, 128.06, 128.92, 133.72, 138.13, 138.75, 149.26, 163.99, 167.20, 168.65.

**2-[5-Methyl-2-(propan-2-yl)phenoxy]-2-oxoethyl 3,4-dihydroxybenzoate (2)** white solid (0.64 g, 1.8 mmol, 75%), ^1^H NMR (400 MHz, Acetone-*d*_6_) δ 1.17 (d, *J* = 6.8 Hz, 6H), 2.28 (s, 3H), 3.12 (m, 1H), 5.14 (s, 2H), 6.90 (d, *J* = 0.8 Hz, 1H), 6.98 (d, *J* = 8.4 Hz, 1H), 7.05 (dd, *J* = 1.2, 8.0 Hz, 1H), 7.24 (d, *J* = 8.0 Hz, 1H), 7.59 (dd, *J* = 2.0, 8.4 Hz, 1H), 7.63 (d, *J* = 2.0 Hz, 1H), 8.62 (s, 2OH). ^13^C NMR (100 MHz, Acetone-*d*_6_) δ 20.65, 23.28, 27.48, 61.62, 115.85, 117.34, 121.68, 123.33, 123.78, 127.15, 127.96, 137.21, 137.88, 145.65, 148.38, 151.28, 166.25, 167.67.

**2-[5-Methyl-2-(propan-2-yl)phenoxy]-2-oxoethyl 3,4,5-trihydroxybenzoate (3)** yellowish white solid (0.79 g, 1.41 mmol, 60%), ^1^H NMR (400 MHz, Acetone-*d*_6_) δ 1.16 (d, *J* = 6.8 Hz, 6H), 2.28 (s, 3H), 2.11 (m, 1H), 5.11 (s, 2H), 6.89 (d, *J* = 0.8 Hz, 1H), 7.06 (dd, *J* = 2.0, 7.6 Hz, 1H), 7.24 (m, 3H). ^13^C NMR (100 MHz, Acetone-*d*_6_) δ 20.86, 23.51, 27.71, 61.88, 110.34, 120.85, 123.59, 127.40, 128.19, 137.47, 138.16, 139.43, 146.31, 148.66, 166.60, 167.90.

**2-[5-Methyl-2-(propan-2-yl)phenoxy]-2-oxoethyl (2E)-3-phenylprop-2-enoate (4)** white solid (0.59 g, 1.7 mmol, 87%), ^1^H NMR (400 MHz, Acetone-*d*_6_) δ 1.24 (d, *J* = 6.8 Hz, 6H), 2.30 (s, 3H), 3.18 (m, 1H), 5.14 (s, 2H), 6.73 (d, *J* = 16.0 Hz, 1H), 6.95 (d, *J* = 2.0 Hz, 1H), 7.09 (dd, *J* = 2.0, 8.0 Hz, 1H), 7.28 (d, *J* = 8.0 Hz, 1H), 7.46 (m, 3H), 7.71 (m, 2H), 7.88 (d, *J* = 16.0 Hz, 1H). ^13^C NMR (100 MHz, Acetone-*d*_6_) δ 21.36, 23.99, 28.16, 62.13, 118.22, 123.98, 127.80, 128.64, 129.71, 130.35, 132.02, 135.57, 137.85, 138.50, 147.37, 149.01, 167.21, 168.13.

**2-[5-Methyl-2-(propan-2-yl)phenoxy]-2-oxoethyl (2E)-3-(4-hydroxyphenyl)prop-2-enoate (5)** white solid (0.65 g, 1.8 mmol, 87%), ^1^H NMR (400 MHz, Acetone-*d*_6_) 1.20 (d, *J* = 6.8 Hz, 6H), 2.30 (s, 3H), 3.15 (m, 1H), 5.08 (s, 2H), 6.52 (d, *J* = 16.0 Hz, 1H), 6.91 (d, *J* = 0.8 Hz, 1H), 6.96 (m, 2H), 7.07 (dd, *J* = 2.0, 8.0 Hz, 1H), 7.26 (d, *J* = 8.0 Hz, 1H), 7.62 (m, 2H), 7.80 (d, *J* = 16.0 Hz, 1H), 8.99 (s, OH). ^13^C NMR (100 MHz, Acetone-*d*_6_) δ 20.89, 23.54, 27.71, 61.55, 114.29, 116.90, 123.56, 126.87, 127.38, 128.19, 131.34, 137.43, 138.11, 147.06, 148.61, 160.98, 167.22, 167.90.

**2-[5-Methyl-2-(propan-2-yl)phenoxy]-2-oxoethyl (2E)-3-(4-chlorophenyl)prop-2-enoate (6)** white solid (0.64 g, 1.7 mmol, 84%), ^1^H NMR (400 MHz, Acetone-*d*_6_), 1.21 (d, *J* = 6.8 Hz, 6H), 2.10 (s, 3H), 3.16 (m, 1H), 5.13 (s, 2H), 6.71 (d, *J* = 16.0 Hz, 1H), 6.93 (d, *J* = 1.6 Hz), 7.08 (dd, *J* = 8.0, 1.6 Hz, 1H), 7.27 (d, *J* = 8.0 Hz, 1H), 7.47 (m, 2H), 7.72 (m, 2H), 7.82 (d, *J* = 16.0 Hz, 1H). ^13^C NMR (100 MHz, Acetone-*d*_6_) δ 21.31, 23.94, 28.12, 62.16, 118.99, 123.93, 127.77, 128.62, 130.44, 131.24, 134.38, 137.30, 137.83, 138.46, 145.83, 148.97, 167.01, 168.07.

**2-[5-Methyl-2-(propan-2-yl)phenoxy]-2-oxoethyl (2E)-3-(2,4-dihydroxyphenyl)prop-2-enoate (7)** white solid (0.57 g, 1.5 mmol, 76%), ^1^H NMR (400 MHz, Acetone-*d*_6_), 1.17 (d, *J* = 6.8 Hz, 6H), 2.25 (s, 3H), 3.11 (m, 1H), 5.03 (s, 2H), 6.49 (dd, *J* = 2.0, 8.4 Hz, 1H), 6.54 (d, *J* = 2.0 Hz, 1H), 6.64 (d, *J* = 16.0 Hz, 1H), 6.87 (d, *J* = 0.8 Hz, 1H), 7.02 (dd, *J* = 0.8, 8.0 Hz, 1H), 7.21 (d, *J* = 8.0 Hz, 1H), 7.48 (d, *J* = 8.0, Hz, 1H), 8.12 (d, *J* = 16.0 Hz, 1H), 8.97 (s, OH), 9.29 (s, OH). ^13^C NMR (100 MHz, Acetone-*d*_6_) δ 19.52, 22.13, 26.29, 60.04, 102.39, 107.84, 112.13, 113.13, 122.10, 125.95, 126.79, 130.37, 135.99, 136.65, 141.70, 147.13, 158.20, 160.71, 166.67, 166.71.

### 2.3. In Vitro Antimalarial Testing of Thymol Derivatives Against Plasmodium Parasites

*Plasmodium knowlesi* A1H1 (originally contributed by Robert W. Moon, London School of Hygiene and Tropical Medicine, UK) was obtained from the Malaria Culture Laboratory, Department of Parasitology, Faculty of Medicine, Universiti Malaya. The *Plasmodium falciparum* 3D7 strain (CQ-sensitive; MRA-102), contributed by Daniel J. Carucci, was sourced from BEI Resources, NIAID, NIH. Both parasites were cultured in fresh human type O+ erythrocytes suspended in RPMI 1640 medium (Invitrogen Life Technologies, Carlsbad, CA, USA) supplemented with 3 g/L glucose, 45 μg/L hypoxanthine, and 50 μg/L gentamicin. The *P. knowlesi* cultures were maintained in RPMI 1640 supplemented with 10% horse serum (Gibco, Auckland, New Zealand), while *P. falciparum* cultures were supplemented with 10% Albumax I, following the method described by Trager and Jensen [27]. Antiplasmodial activity of thymol derivatives was evaluated using a modified Plasmodium lactate dehydrogenase (pLDH) assay based on the protocol by Makler and Hinrichs [28]. Synchronized parasites (2% hematocrit, 2% parasitemia) were seeded in 96-well plates. Thymol derivatives in varying concentrations (100 to 0.001 µM) were added to each well containing 99 μL of parasite culture. Untreated parasitized red blood cells served as negative controls, while non-parasitized red blood cells acted as blanks. Chloroquine diphosphate was used as a positive control. Plates were incubated under a gas mixture (5% O_2_, 5% CO_2_, 90% N_2_) at 37 °C for 24 h (*P. knowlesi*) and 48 h (*P. falciparum*). After incubation, plates were frozen at −20 °C for 24 h, followed by thawing at 37 °C for 30 min to lyse red blood cells. Subsequently, 25 μL of the lysate was transferred to a new plate, followed by the addition of 100 μL Malstat reagent and 25 μL of nitro blue tetrazolium/phenazine ethosulfate (NBT/PES) solution. Absorbance was measured at 650 nm using a SpectraMax Paradigm^®^ Multi-Mode microplate reader (Agilent Technologies, Santa Clara, CA, USA). EC_50_ values (half-maximal effective concentration) were calculated using non-linear regression analysis in GraphPad Prism 8 software.

### 2.4. Cytotoxicity Assessment of Thymol Derivatives on Human Cardiac Fibroblasts

Human cardiac fibroblasts (HCF; Catalog No. 6300) were obtained from ScienCell Research Laboratories (Carlsbad, CA, USA). HCF are normal mammalian cells found in the heart. The cytotoxic effects of thymol derivatives on HCF were evaluated using the 3-(4,5-dimethylthiazol-2-yl)-2,5-diphenyltetrazolium bromide (MTT) assay, adapted from established protocols [29]. HCF (2 × 10^5^ cells/well) were seeded into 96-well plates in 100 μL of complete medium and incubated for 24 h to allow for cell adherence. The medium was then replaced with 100 μL of serum free-medium, followed by treatment with varying concentrations of thymol derivatives (0.2–3.0 μM) for 48 h. Untreated cells and vehicle controls (0.01% DMSO) were included for comparison. Following the treatment period, 10 μL of MTT solution (5 mg/mL) was added to each well and incubated for 3–4 h at 37 °C in the dark to allow the formation of purple formazan crystals in viable cells. The supernatant was carefully removed, and 100 μL of dimethyl sulfoxide (DMSO) was added to dissolve the crystals. After 15 min of incubation at room temperature, absorbance was measured at 570 nm using an ELISA microplate reader. Cell viability (%) was calculated relative to the untreated control. Dose–response curves were generated using GraphPad Prism 8 software with non-linear regression analysis to determine the half-maximal cytotoxic concentration (CC_50_) of thymol derivatives.

$$Cell\;viability\left(\%\right)=\frac{Absorbance\;of\;treated\;cells}{Absorbance\;of\;control}\times 100\%$$

### 2.5. Selectivity Indexes Calculation

The selectivity index was determined by calculating the ratio between the CC_50_ value for human cardiac fibroblasts (HCF) and the EC_50_ values for *Plasmodium knowlesi* or *P. falciparum*. The SI was calculated using the formula:

$$Selectivity\;Index\left(SI\right)=\frac{{CC}_{50}(HCF)}{{EC}_{50}(Plasmodium\;spp.)}$$

A higher SI value (SI > 10) suggests that the test compound exhibits selective toxicity toward the parasite with minimal cytotoxic effects on normal human cells, indicating potential efficacy and safety. Compounds with an SI greater than 10 (SI > 10) were classified as highly selective antimalarial agents [30].

### 2.6. Molecular Docking Analysis

**Figure 1:**
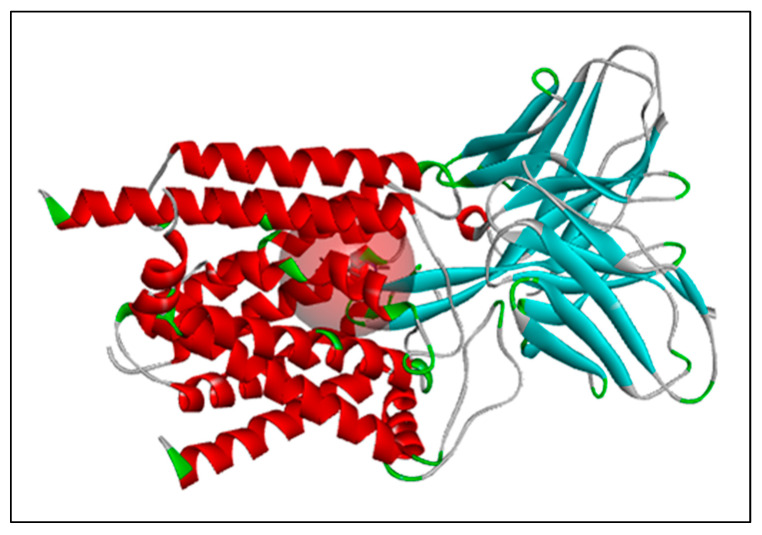
The chosen target protein PfCRT, with its active site indicated by a red sphere. Indicators: Red indicates α-helices, cyan β-sheets, green loops, grey coils, and the pink region highlights the binding pocket.

To investigate the binding interactions of thymol derivatives (ligands) with PfCRT was selected as the target receptor. PfCRT is chosen as the docking target because it plays a central role in mediating resistance to 4-aminoquinoline antimalarials and remains one of the most relevant transport proteins in the context of drug-resistant *P. falciparum* [9]. The three-dimensional structure of PfCRT (PDB ID: 6UKJ) was obtained from the Protein Data Bank (https://www.rcsb.org/structure/6UKJ, accessed on 1 January 2025). The 3D conformations of thymol derivatives were generated using Marvin Sketch version 1.5.6 and further refined with the ligand preparation wizard in the Schrödinger Small Molecule Drug Discovery Suite 2017-1. Prior to docking, all water molecules were removed from the PfCRT structure, while hydrogen atoms and charges were added according to standard geometries. The ligand-binding site was defined within the transmembrane domain 1 of PfCRT (Figure 1), with grid box dimensions of 30 × 30 × 30 and coordinates x = 149.5, y = 169.9, and z = 138.9, as reported by Patowary et al. [31]. Molecular docking was performed using AutoDock Vina (https://vina.scripps.edu/, accessed on 1 January 2025), allowing ligand flexibility while maintaining the receptor in a rigid conformation. The resulting docked poses were evaluated based on their binding interactions. To ensure reliability, the docking protocol was validated through redocking of a reference ligand into the PfCRT binding pocket, following the approach of Rao et al. [32]. CQ was used as a reference drug in the docking study to evaluate the compounds. Docking scores reflecting the binding affinity of each compound to PfCRT were calculated and subsequently compared with those of CQ [33,34].

### 2.7. ADMET Studies of Thymol Derivatives

The physicochemical and pharmacokinetic properties, including absorption, distribution, metabolism, excretion, and toxicology (ADMET), were predicted using the online platforms admetSAR 2.0 (http://lmmd.ecust.edu.cn/admetsar2/admetopt/, accessed on 1 January 2025) [35], SWISS ADME (http://www.swissadme.ch/index.php, accessed on 1 January 2025) [36], and ADMETlab 2.0 (https://admetmesh.scbdd.com/service/evaluation/index, accessed on 1 January 2025) [37]. The in silico analyses provide valuable insights into the potential pharmacological behavior of the compounds within biological systems, including blood, tissues, and organs, and serve as an efficient strategy for prioritizing and screening large libraries of candidate molecules. Drug-likeness was assessed using Lipinski’s “Rule of Five,” which evaluates critical molecular descriptors: molecular weight (MW) < 500 g/moL, hydrogen bond acceptors (nHA) ≤ 10, hydrogen bond donors (nHD) ≤ 5, and the logarithm of the n-octanol/water partition coefficient (logP) ≤ 5 [38]. Compounds with two or more violations of these criteria are predicted to exhibit poor oral bioavailability and are generally considered unsuitable for development as drug candidates.

### 2.8. Statistical Analysis

Statistical analyses were performed using ANOVA. Results are presented as mean ± standard deviation (SD), and differences were considered statistically significant at *p* < 0.05.

## 3. Results

### 3.1. Synthesis of Thymol Derivatives

**Scheme 1:**
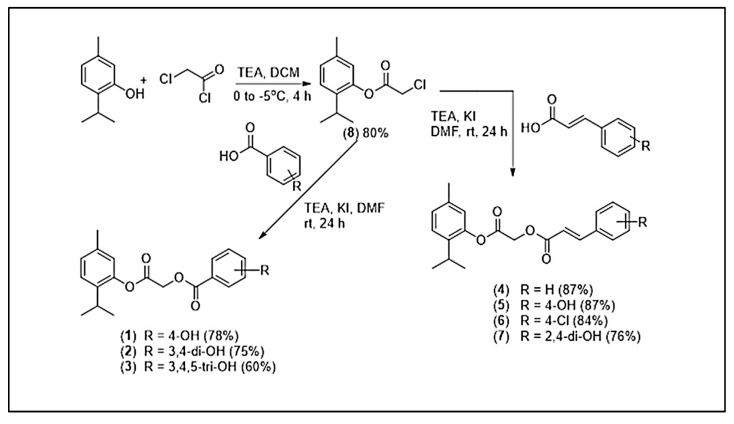
Synthetic scheme for compounds **1**–**7**.

Seven thymol-based derivatives were designed and structurally characterized. As reported in previous studies, the syntheses of compounds **1**, **2**, **4**, **5**, and **6** have been described in the literature [23,24]. In the present work, these compounds were re-synthesized and their cytotoxic activities against *Plasmodium* strains were re-evaluated to provide a consistent basis for structure–activity relationship (SAR) analysis. In addition, two previously unreported derivatives, compounds **3** and **7**, were synthesized using the same synthetic approach to obtain a more comprehensive SAR profile. All compounds were prepared via a two-step reaction sequence, as outlined in Scheme 1. The parent molecule, thymol, was chemically modified through esterification and conjugation with hydroxybenzoic acid and cinnamic acid derivatives to generate analogs with varied substitution patterns. The resulting derivatives included hydroxybenzoate conjugates: (**1**) 4-OHBT, (**2**) 3,4-OHBT, and (**3**) GAT, as well as cinnamate derivatives: (**4**) CT, (**5**) 4-OHCT, (**6**) CCT, and (**7**) 2,4-OHCT. These structural modifications introduce functional groups, such as hydroxy (–OH) groups in varying numbers and chlorine (Cl) substituents, which are known to influence physicochemical properties, biological activity, and pharmacokinetic behavior.

### 3.2. Antiplasmodial and Cytotoxic Activities

**Figure 2:**
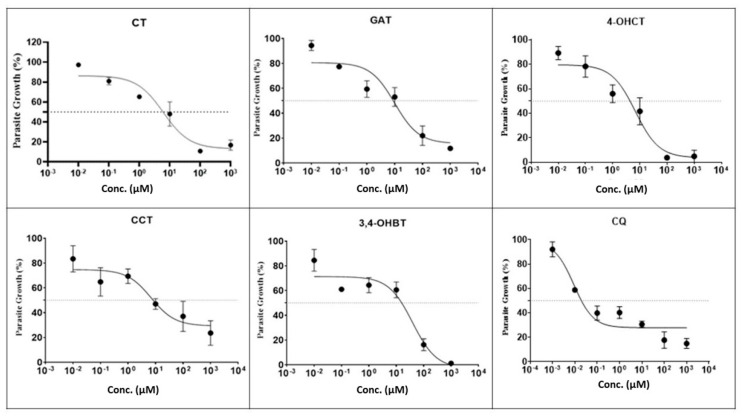
Effect of thymol derivatives against *P. falciparum* 3D7 (CQ-sensitive). Indicator: The middle horizontal line represents 50% parasite growth.

The results in Table 1 summarize the in vitro antiplasmodial activity, cytotoxicity, and selectivity of thymol and its derivatives against *Plasmodium falciparum* (3D7 and K1 strains) and *Plasmodium knowlesi* A1H1. Several derivatives exhibited enhanced potency compared to the parent compound, thymol, which showed EC_50_ values of 15.1 ± 0.5 µM (3D7), 34.9 ± 11.5 µM (K1), and 18.8 ± 8.1 µM (A1H1), with selectivity indexes (SI) of 6.6, 2.8, and 5.3, respectively. Among the thymol derivatives, compound **4** demonstrated the strongest activity against 3D7 (EC_50_ = 6.01 ± 1.7 µM), with favorable SI values of 16.5. Compound **6** also exhibited notable potency against 3D7 and K1 with EC_50_ = 6.8 ± 1.1 µM and 17.6 ± 0.3 µM, respectively as well as high SI values (14.6 and 5.6, respectively), indicating improved selectivity relative to thymol. Additionally, compound **5** and compound **3** displayed strong activities against 3D7 (EC_50_ = 6.8 ± 1.1 µM and 9.9 ± 0.4 µM respectively). In contrast, compound **1** exhibited weaker activity, with higher EC_50_ values (>16 µM) and lower SI values (<6), indicating limited selectivity. Antimalarial activity was categorized based on established thresholds i.e., compounds with EC_50_ values < 1 µM were considered highly potent, those within 2–20 µM as active, 21–100 µM as moderately active, 101–200 µM as weakly active, and >201 µM as inactive [39,40]. Dose–response inhibition profiles of thymol derivatives (**2**) 3,4-OHBT, (**3**) GAT, (**4**) CT, (**5**) 4-OHCT, and (**6**) CCT against *P. falciparum* 3D7 (CQ-sensitive). Parasite growth (%) decreased in a concentration-dependent manner for all compounds, with sigmoidal curve fitting used to derive EC_50_ values. Among the derivatives, (**5**) 4-OHCT and (**2**) 3,4-OHBT showed steeper inhibition curves, indicating higher potency, whereas (**4**) CT and (**6**) CCT exhibited more gradual declines in parasite growth. CQ served as the positive control and demonstrated the expected strong inhibitory activity at low micromolar to sub-micromolar concentrations (Figure 2). For comparison, the reference drug CQ consistently demonstrated EC_50_ values below 1 µM across all tested Plasmodium strains, confirming its strong potency and superior efficacy relative to thymol derivatives.

The cytotoxicity evaluation against HCF mammalian cells revealed that most thymol derivatives were non-toxic, with CC_50_ values greater than 99 µM, except for compound **3** (CC_50_ = 71.4 ± 4.5 µM) and compound **1** (CC_50_ = 58.4 ± 2.3 µM), which exhibited moderate cytotoxic effects. Importantly, the majority of compounds demonstrated favorable selectivity indexes (SI), suggesting preferential activity against *Plasmodium* parasites over mammalian cells. These results highlight the therapeutic potential of selected derivatives, especially compound **4**, **5**, and **6**, as they combine strong antiplasmodial activity with favorable safety margins. Overall, these findings suggest that structural modifications of thymol particularly hydroxylation and cinnamate conjugation enhanced antiplasmodial activity and selectivity.

### 3.3. In Silico Molecular Docking

Molecular docking analysis of thymol and its derivatives against PfCRT revealed varied binding affinities and inhibition constants. Among the derivatives, compound **3** exhibited the strongest binding affinity (−8.4 kcal/mol) and the lowest inhibition constant (*K*i = 0.7 µM), forming hydrogen bonds with Asn209, Glu207, and Phe203 (Table 2). Compound **4** and **6** showed similar high binding affinities (−8.3 kcal/mol) with low *K*i values (0.8 µM), although no hydrogen bond interactions were observed. Other derivatives, including compound **1**, **2**, **5**, and **7**, demonstrated moderate binding affinities (−7.2 to −8.0 kcal/mol) and formed hydrogen bonds with specific amino acids such as Tyr110, Arg101, Ser220, and Tyr345. Thymol exhibited the weakest binding affinity (−6.7 kcal/mol) with a high *K*i value (12.3 µM), interacting via hydrogen bonds with Ser202 and Trp95. Overall, these results indicate that certain thymol derivatives, particularly compound **3**, **4**, and **6** have enhanced binding and inhibitory potential against PfCRT compared to the parent compound.

**Figure 3:**
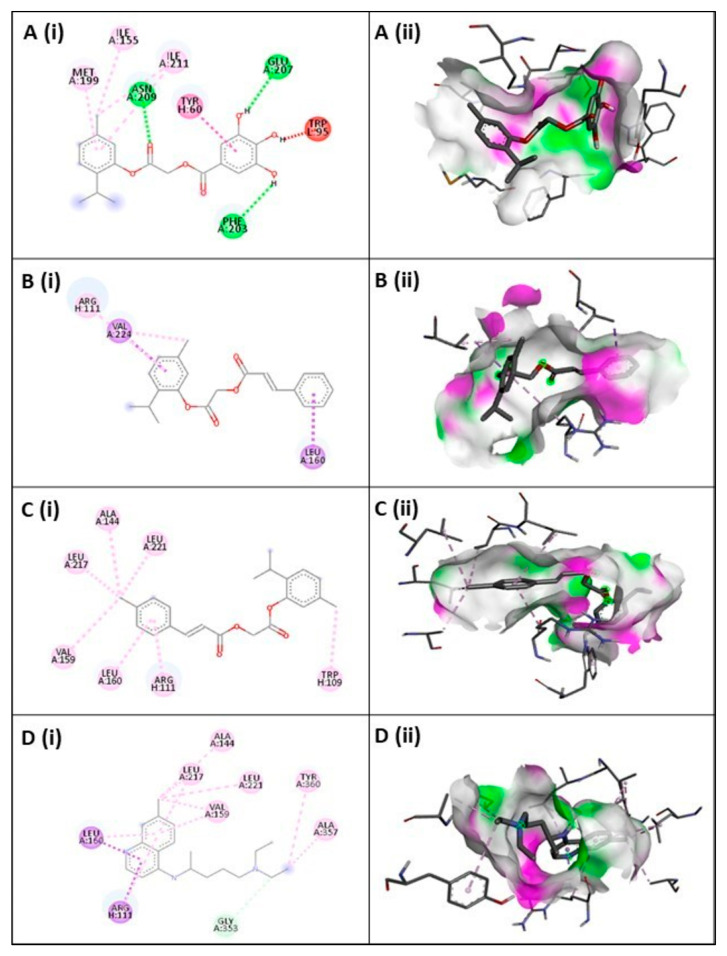
2D (**i**) and 3D (**ii**) representations of ligand–protein interactions within the binding pocket for Compound **3** (**A**), Compound **4** (**B**), Compound **6** (**C**), and CQ (**D**). In 2D visualization, green or pink denote hydrogen bond acceptor or donor residues, red indicates steric clashes, and other colors represent non-covalent interactions such as hydrophobic, aromatic, or electrostatic contacts. In 3D visualization, green regions indicate H-bond donors and pink regions show H-bond acceptors.

The molecular docking of compound **3** with PfCRT protein revealed a binding affinity of −8.4 kcal/mol with an inhibition constant (*K*i) of 0.7 μM, indicating strong binding potential. The 2D interaction diagram (Figure 3) showed that compound **3** formed key hydrogen bonds with Asn209, Glu207, and Phe203 (green dashed lines), which play an essential role in stabilizing the ligand within the PfCRT binding cavity. Additionally, hydrophobic interactions were observed with Met199, Ile155, Ile211, Tyr60, and Trp95 (pink dashed lines), further contributing to ligand stabilization. The 3D docking visualization confirmed that compound **3** fits well within the hydrophobic pocket of PfCRT, with complementary hydrophilic and hydrophobic surface regions highlighted in green and magenta. Collectively, these findings suggest that compound **3** exhibits strong and stable binding to PfCRT, mediated by a combination of hydrogen bonding and hydrophobic interactions, which may underlie its inhibitory activity against the protein.

### 3.4. ADMET Properties

The ADMET predictions (Table 3) revealed favorable pharmacokinetic and safety characteristics for the thymol derivatives compared with CQ. In terms of absorption, all compounds exhibited high human intestinal absorption (HIA > 0.61), with compound **4** and CQ showing the strongest probability (0.9888 and 0.9939, respectively). Caco-2 permeability was moderate across the derivatives (0.50–0.74), while blood–brain barrier (BBB) penetration was highest for compound **4** and compound **3** (>0.81), indicating potential central nervous system exposure. Most derivatives were predicted as P-glycoprotein inhibitors, suggesting possible influence on drug efflux mechanisms.

For distribution, subcellular localization was predominantly mitochondrial (probability > 0.87), supporting intracellular bioavailability. Metabolic profiling showed that all derivatives are substrates for CYP3A4 but non-substrates for CYP2C9 and CYP2D6. Inhibition patterns varied: compound **4** strongly inhibited CYP1A2 (0.9225), CYP2C9 (0.8854), and CYP2C19 (0.8952). Excretion data indicated moderate half-lives (6.8–11.9 h) and clearance values ranging from 0.134 to 0.950 mL/min/kg, suggesting acceptable elimination profiles. Toxicity assessment predicted non-AMES mutagenicity and non-carcinogenicity across all compounds, with weak inhibition of the hERG channel (0.96–0.98), implying low cardiotoxicity risk. However, high ecological toxicity (fish, Tetrahymena, and honeybee) and poor biodegradability were observed, indicating environmental concerns. Acute oral toxicity fell into category III, consistent with moderate safety margins. Overall, thymol derivatives demonstrated drug-like properties with acceptable ADMET characteristics, particularly favorable absorption, non-carcinogenicity, and manageable toxicity profiles. These findings support their potential as lead scaffolds for further optimization as antimalarial agents.

## 4. Discussion

The present study demonstrated that chemical modification of thymol (Scheme 1) through esterification and conjugation with hydroxybenzoic acid and cinnamic acid derivatives significantly improved antiplasmodial activity and selectivity. Several derivatives, particularly compound **3**, **4**, **5**, and **6** displayed superior efficacy compared to the parent thymol, which exhibited only moderate activity (EC_50_ = 6.01–34.91 µM across parasite strains). Compound **4** was the most potent candidate, showing an EC_50_ of 6.01 µM against *P. falciparum* 3D7, with a high selectivity index (SI = 16.5), suggesting favorable therapeutic potential (Figure 2). These findings are consistent with earlier reports that structural modifications such as hydroxylation, halogenation, and hybridization enhance the antimalarial activity of thymol analogs [21,24].

The cinnamate derivatives, especially compound **4** and **6** showed broad-spectrum activity against both CQ-sensitive and -resistant strains, supporting the role of cinnamic acid conjugation in improving bioactivity. Similar enhancements have been observed in other natural product derivatives, where phenolic hydroxyl and cinnamate moieties contributed to stronger binding affinity and redox properties [24]. Molecular docking further supported these experimental findings. Compound **3** exhibited strong binding affinities to PfCRT (−8.4 kcal/mol), through hydrogen bonding with Asn209, Glu207, Gln352, and Thr76, alongside hydrophobic stabilization by residues such as Met199, Leu217, and Phe203. These interactions are in agreement with previous structural analyses showing that PfCRT mutations affect ligand recognition and drug resistance [29]. The ability of thymol derivatives to form stable complexes with PfCRT underscores their potential in overcoming CQ resistance mechanisms [30]. ADMET predictions revealed favorable pharmacokinetic properties across the series, with high intestinal absorption and acceptable elimination half-lives. Notably, compound **3** and **4** demonstrated high BBB penetration (>0.81), suggesting potential utility in addressing cerebral malaria. These results are consistent with recent efforts to optimize phytochemical derivatives for improved metabolic stability and safety [40].

**Figure 4:**
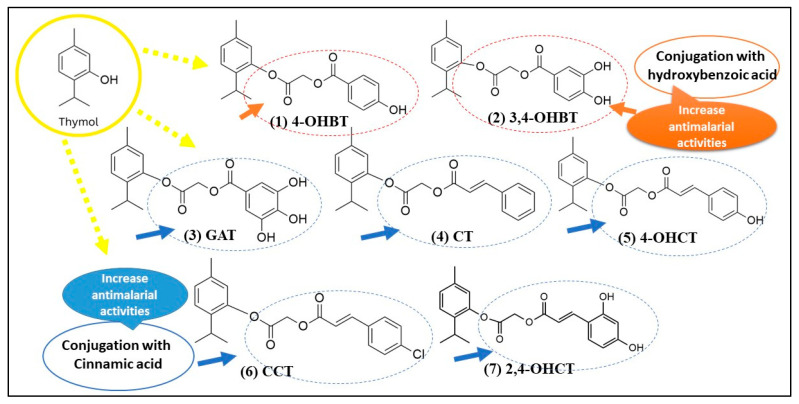
Structural modification of thymol via hydroxybenzoic and cinnamic acid conjugation enhances antimalarial activity.

The conjugation of thymol with hydroxybenzoic acids (compound **1** and **2**), as well as cinnamic acid conjugates could increase antimalarial activity versus thymol alone (Figure 4). Cinnamic acid (and derivatives) conjugated to known antimalarial scaffolds or drugs can yield enhanced or dual-mode activity [41]. Also, hydroxybenzoic acid derivatives (especially those with multiple phenolic (hydroxyl) groups) have good antiplasmodial activity. One compound, methyl 4-benzoxy-3,5-dihydroxybenzoate, is singled out as a potential lead because of fairly strong potency and good water solubility (≈3.72 mM) [42].

The conjugating thymol to hydroxybenzoic acid or cinnamic acid could improve or modulate antimalarial activity by increasing polarity, solubility and distribution, adding phenolic or antioxidant functions, dual-mode or synergistic interactions and potential to evade resistance. The addition of carboxylates or hydroxylated aromatic acids may help with solubility, cell uptake or targeting for example, allowing better distribution in parasitized erythrocytes or easier crossing of membranes. Hydroxybenzoic acids and cinnamic acids themselves often have antioxidant or redox-modulating activity. Since some antimalarials (or their derivatives) act via generating oxidative stress or interfering with parasite redox systems (such as with 4-chlorothymol), having both parts may produce additive or synergistic redox perturbation. The conjugate may combine the mode(s) of action of thymol (membrane interactions, phenolic oxidation, etc.) with those of the acid moiety (binding to certain enzymes, antioxidant or pro-oxidant behavior, or interactions with parasite targets) or improve pharmacokinetics.

Novel conjugates with new scaffolds (i.e., not simply quinoline or artemisinin analogues) may have less cross-resistance; also, modulating metabolism (e.g., inhibiting cytochrome P450 or being less readily metabolized) can help prolong activity. Compound OHCT, for example, inhibits human CYP3A4, which degrades some antimalarial drugs; thus OHCT potentially increases half-life of partner drugs [43]. This the first reported antimalarial of thymol conjugated to 4-hydroxybenzoic acid (compound **1**) or combinations such as compound **5** (i.e., combining thymol with cinnamic acid and terpenone). The position of hydroxyls on the benzoic acid (ortho, meta, para) matters, as does whether the acid moiety is free (COOH), esterified, or linked via some spacer. The stereochemistry, hydrophobic vs. hydrophilic balance, size, affect cell uptake, stability, toxicity and effective concentration in parasite infected erythrocytes [44,45].

In this study, thymol and its derivatives possess hydrophobic phenolic scaffolds capable of interacting with lipid membranes and transporter proteins. One potential molecular target relevant to antimalarial resistance is the PfCRT, a transmembrane protein located in the digestive vacuole membrane of the parasite. PfCRT mutations (particularly K76T and others along transmembrane helices 1–10) enable efflux of protonated CQ and related cationic drugs from the parasite’s digestive vacuole, leading to CQ resistance [46,47]. Compounds that can interfere with PfCRT-mediated efflux or alter vacuolar membrane permeability may thus restore or enhance sensitivity to quinoline-based antimalarials. The phenolic and lipophilic nature of thymol and its conjugates (e.g., Compound **1**, **2**, **5**, and **6**) allows them to integrate into lipid bilayers and disrupt transporter function. Two mechanisms are plausible, PfCRT modulation or inhibition. Molecular docking studies with similar small lipophilic phenols (e.g., eugenol, carvacrol) have shown possible binding within PfCRT’s transmembrane cavity, interfering with substrate translocation. The aromatic–hydroxylated substituents in hydroxybenzoic and cinnamic acid moieties may enhance hydrogen bonding with PfCRT’s polar residues (e.g., Lys76, Thr93, Asn326), potentially blocking drug efflux channels. Thymol conjugates can alter digestive vacuole redox balance and membrane potential, indirectly impairing PfCRT function, similar to how 4-chlorothymol induces oxidative stress and disrupts parasite ion homeostasis [17]. Such dual activity, membrane perturbation and PfCRT interference could overcome CQ resistance by increasing intra-vacuolar accumulation of CQ or related drugs. Cinnamic acid conjugates have been reported to enhance CQ uptake in resistant *P. falciparum* strains by modulating vacuolar pH and possibly PfCRT function [41]. Phenolic acids (including hydroxybenzoic derivatives) act as weak bases and membrane disruptors that alter proton gradients factors crucial to PfCRT-driven efflux mechanisms. In silico models indicate that PfCRT-binding inhibitors often feature aromatic, hydroxylated, and moderately lipophilic pharmacophores consistent with the designed thymol conjugates in this study.

## 5. Conclusions

The present findings demonstrate that the evaluated thymol derivatives generally exhibited favorable antimalarial inhibitory activity against both chloroquine (CQ)-sensitive and CQ-resistant *P. falciparum* strains, as well as *P. knowlesi*, while displaying low cytotoxicity toward mammalian cells. Among the synthesized derivatives, compounds **4**, **5**, and **6** exhibited superior antimalarial potency and safety profiles compared with thymol, highlighting their potential as lead candidates. Molecular docking analyses revealed strong and stable interactions between compound **4** and PfCRT, suggesting its potential to target the PfCRT resistance-associated protein. Furthermore, ADMET predictions for compound **4** supported favorable pharmacokinetic and safety profiles, including acceptable absorption, metabolism, and clearance characteristics. Overall, this study provides valuable insights into the antimalarial potential of thymol derivatives and contributes to the discovery and development of potent antimalarial agents.

## Figures and Tables

**Table 1:** In vitro Antimalarial Activity, Cytotoxicity, and Selectivity Indexes of Thymol Derivatives.

		Antimalarial Assay (pLDH)			Cytotoxic Test (MTT)	Selectivity Indexes (SI) = (MTT CC_50_/pLDH EC_50_)		
No	Compounds	EC_50_ ± SD Against *Pf*3D7 (µM)	EC_50_ ± SD Against *Pf*K1 (µM)	EC_50_ ± SD Against *Pk*A1H1 (µM)	CC_50_ ± SD Against HCF Cell Line (µM)	SI *Pf*3D7	SI *Pf*K1	SI *Pk*A1H1
1	Thymol	15.1 ± 0.5	34.9 ± 11.5	18.8 ± 8.1	>99	6.6	2.8	5.3
2	4-OHBT (**1**)	16.7 ± 8.3	22.2 ± 1.3	30.2 ± 2.3	58.4 ± 2.3	3.5	2.6	1.9
3	3,4-OHBT (**2**)	11.9 ± 2.3	20.6 ± 2.9	22.8 ± 7.1	>99	8.3	4.8	4.3
4	GAT (**3**)	9.9 ± 0.4	11.3 ± 3.7	16.5 ± 6.1	71.4 ± 4.5	7.2	6.3	4.3
5	CT (**4**)	6.01 ± 1.7	30.9 ± 25.5	41.4 ± 8.1	>99	16.5	3.2	2.4
6	4-OHCT (**5**)	6.8 ± 1.1	16.2 ± 0.2	28.3 ± 6.5	>99	14.6	6.1	3.5
7	CCT (**6**)	6.8 ± 1.1	17.6 ± 0.3	31.3 ± 12.7	>99	14.6	5.6	3.2
8	2,4-OHCT (**7**)	10.9 ± 2.3	24.1 ± 0.6	20.5 ± 6.7	52.4 ± 3.4	4.8	2.2	2.6
9	CQ	0.008 ± 0.001	0.50 ± 0.01	0.001 ± 0.0005	98.90 ± 7.97	12,362	197	98,900

Note: Threshold level: <1 µM: Potent; 1–20 µM: Active/Good; 21–100 µM: Moderately Active; 101–200 µM: Weak; >201: Inactive µM.

**Table 2:** Binding Affinity, Inhibition Constants (*K*i), and Hydrogen Bond Interactions of Thymol Derivatives with PfCRT.

Compounds	Binding Affinity (kcal/moL)	Inhibition Constant, *K*i Value (µM)	Hydrogen Bond, H-Bond (Interaction with Amino Acids)
4-OHBT (**1**)	−7.7	2.3	Tyr110
3,4-OHBT (**2**)	−7.2	5.3	Arg101
GAT (**3**)	−8.4	0.7	Asn209, Glu207, Phe203
CT (**4**)	−8.3	0.8	-
4-OHCT (**5**)	−7.9	1.6	Ser220, Tyr345
CCT (**6**)	−8.3	0.8	-
2,4-OHCT (**7**)	−8.0	1.4	-
Thymol	−6.7	12.3	Ser202, Trp95

**Table 3:** ADMET Profiling of Thymol Derivatives.

Compound ID	CT (4)	GAT (3)	CCT (6)	CQ
Parameters	A (Absorption)			
Blood–Brain Barrier (BBB) (cm s^−1^)	0.8153	0.8171	0.8919	0.7421
Human Intestinal Absorption (HIA)	0.9888	0.6127	0.9901	0.9939
Caco-2 Permeability (log cm s^−1^)	0.7412	0.6554	0.7088	0.5804
P-glycoprotein Substrate	0.5661	0.5654	0.5810	0.8000
P-glycoprotein Inhibitor	0.8273	0.6819	0.6633	0.6220
Renal Organic Cation Transporter	0.8516	0.9019	0.8586	0.6046
	D (Distribution)			
Subcellular localization Mitochondria	0.9319	0.8971	0.9132	0.8743
	M (Metabolism)			
CYP450 2C9 Substrate	0.7555	0.7773	0.7625	0.8422
CYP450 2D6 Substrate	0.8533	0.8852	0.8810	0.8804
CYP450 3A4 Substrate	0.6026	0.5157	0.6468	0.6009
CYP450 1A2 Inhibitor	0.9225	0.6726	0.8405	0.8586
CYP450 2C9 Inhibitor	0.8854	0.5557	0.8417	0.9071
CYP450 2D6 Inhibitor	0.8346	0.8763	0.9102	0.9218
CYP450 2C19 Inhibitor	0.8952	0.7221	0.8395	0.9025
CYP450 3A4 Inhibitor	0.5427	0.8105	0.5337	0.8308
CYP Inhibitory Promiscuity	0.7680	0.8154	0.7846	0.5496
	E (Excretion)			
Half-time (T_1/2_)	0.734	0.779	0.368	0.134
Clearance (CL, mL/min/kg)	6.818	8.124	5.398	6.818
	T (Toxicity)			
Human Ether-a-go-go-Related Gene Inhibition	0.9659	0.9810	0.9530	0.6959
AMES Toxicity	0.7574	0.8521	0.8207	0.9106
Carcinogens	0.7968	0.8651	0.7374	0.8374
Fish Toxicity	0.9943	0.9831	0.9983	0.9911
Tetrahymena Pyriformis Toxicity	0.9992	0.9975	0.9999	0.9987
Honey Bee Toxicity	0.7712	0.6557	0.7228	0.8583
Biodegradation	0.6889	0.7451	0.9627	1.0000
Acute Oral Toxicity (kg mol^−1^)	0.6010	0.7781	0.7101	0.7370
Carcinogenicity	0.6301	0.7402	0.5931	0.6847

Indicators: Non-unit values represent the probability associated with each respective parameter.

## Data Availability

The data presented in this study are available in this article.

## Supplementary Materials

The following supporting information can be downloaded at: https://www.mdpi.com/article/10.3390/biomedicines14010123/s1.

## Abbreviations

The following abbreviations are used in this manuscript:

*P. falciparum*	*Plasmodium falciparum*
*P. knowlesi*	*Plasmodium knowlesi*
PfCRT	*Plasmodium falciparum* chloroquine resistant transporter
EC_50_	half maximal effective concentration
pLDH	plasmodium lactate dehydrogenase
ELISA	enzyme-linked immunosorbent assay
MTT assay	3-(4,5-dimethylthiazol-2-yl)-2,5-diphenyltetrazolium bromide assay
