# Supplementary material for: Thymol Derivatives as Antimalarial Agents: Synthesis, Activity Against Plasmodium falciparum, ADMET Profiling, and Molecular Docking Insights
Source: Biomedicines. 2026 Jan 8;14(1):123. doi: 10.3390/biomedicines14010123 (PMC12839381; doi:10.3390/biomedicines14010123)
Supplement: Supplementary file 1 [file biomedicines-14-00123-s001.zip › biomedicines-3972112-supplementary.pdf]

Supplementary Data

Compound **8 (H-NMR)**

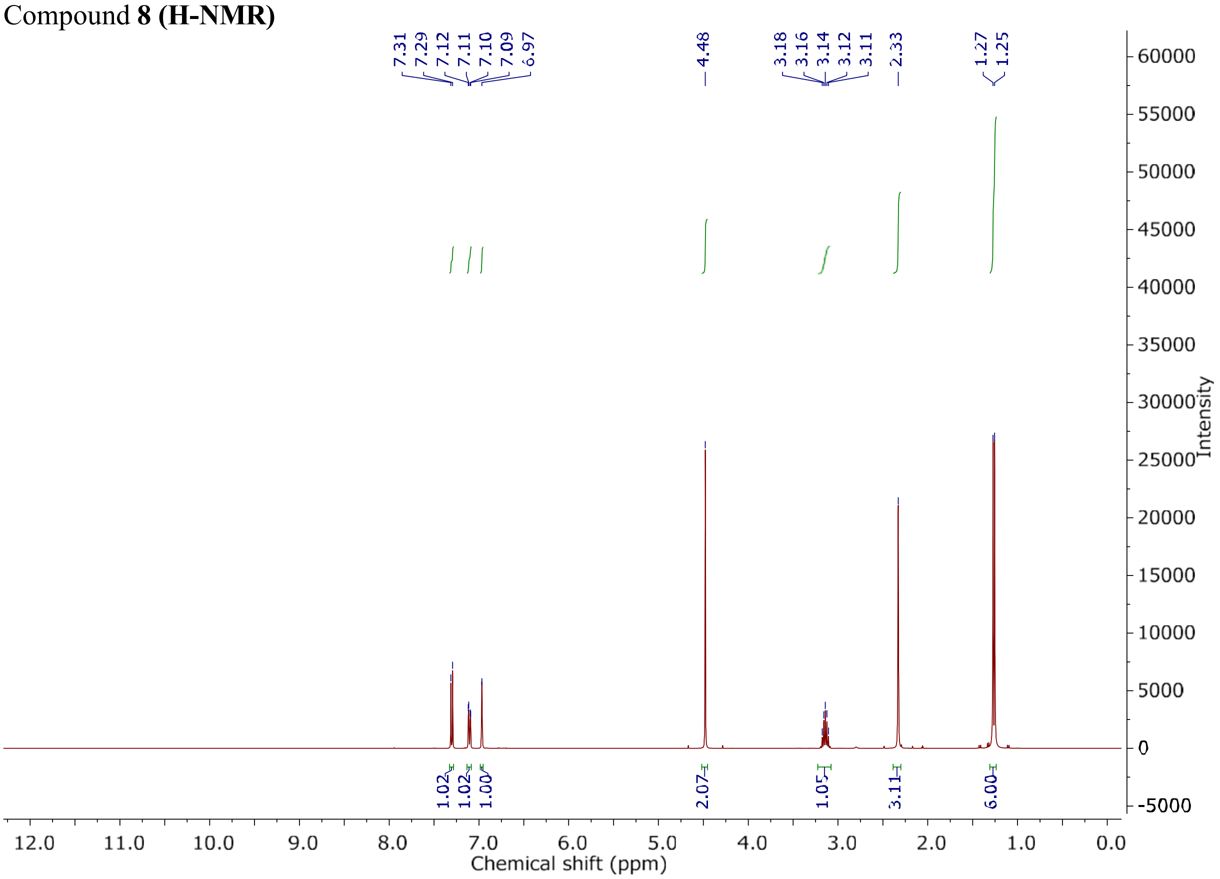

Compound **1** (4-OHBT)

H-NMR

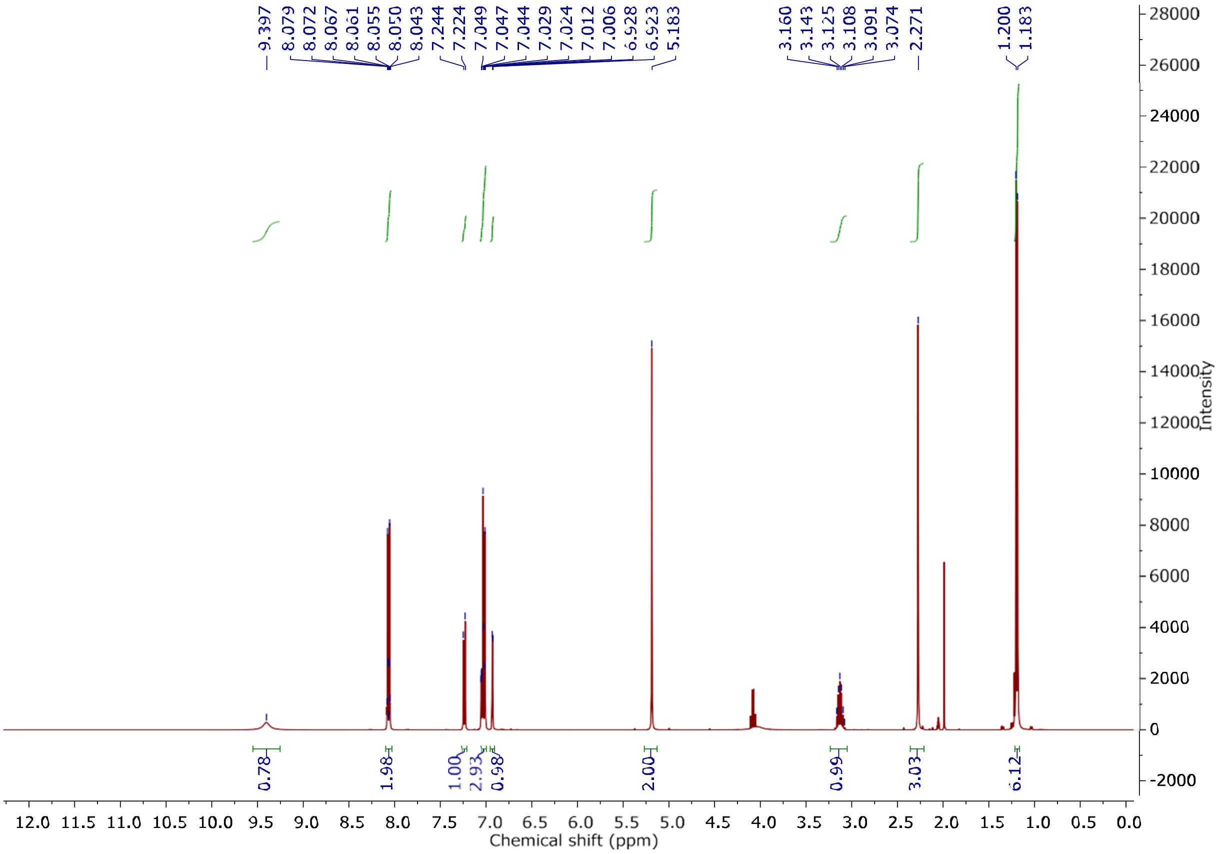

(C-NMR)

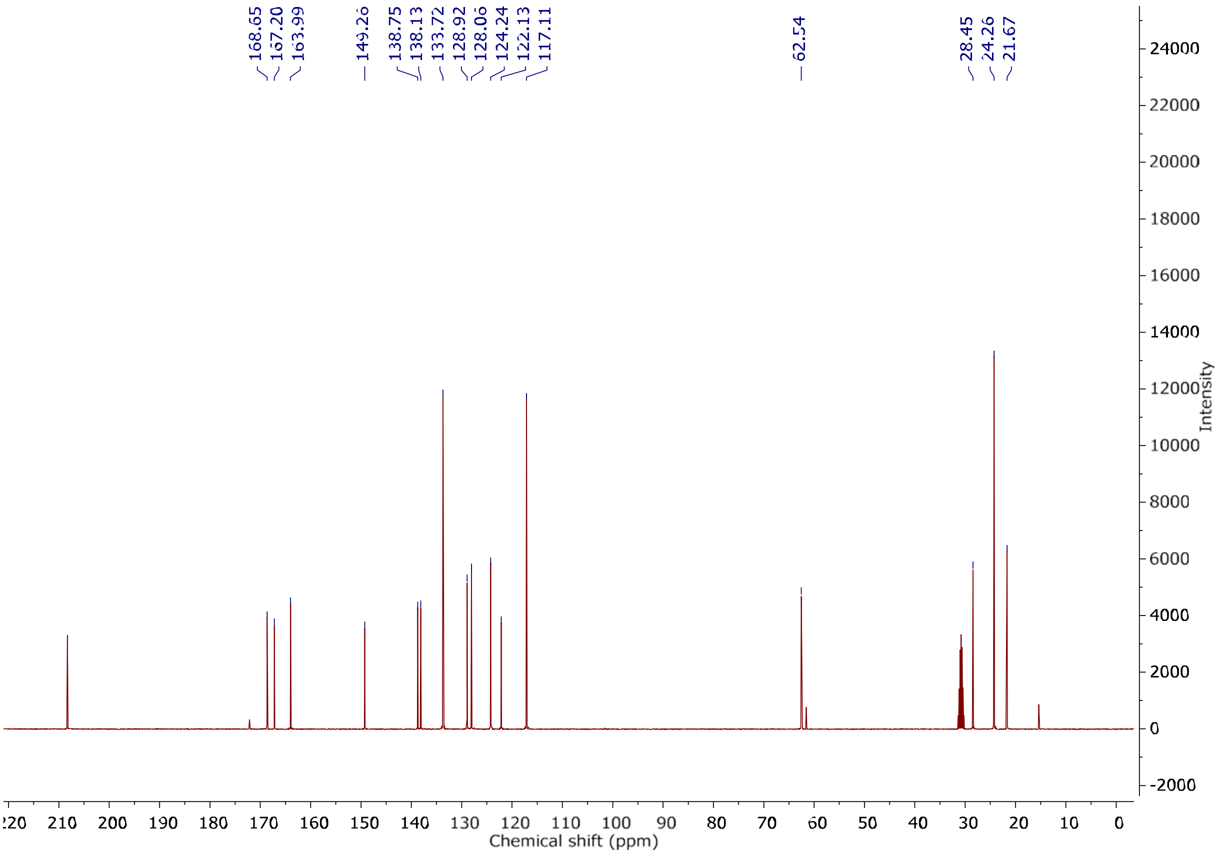

168.65
167.20
163.99
149.26
138.75
138.13
133.72
128.92
128.06
124.24
122.13
117.11
62.54
28.45
24.26
21.67
Intensity
Chemical shift (ppm)

Compound **2** (3,4-OHBT)
(H-NMR)

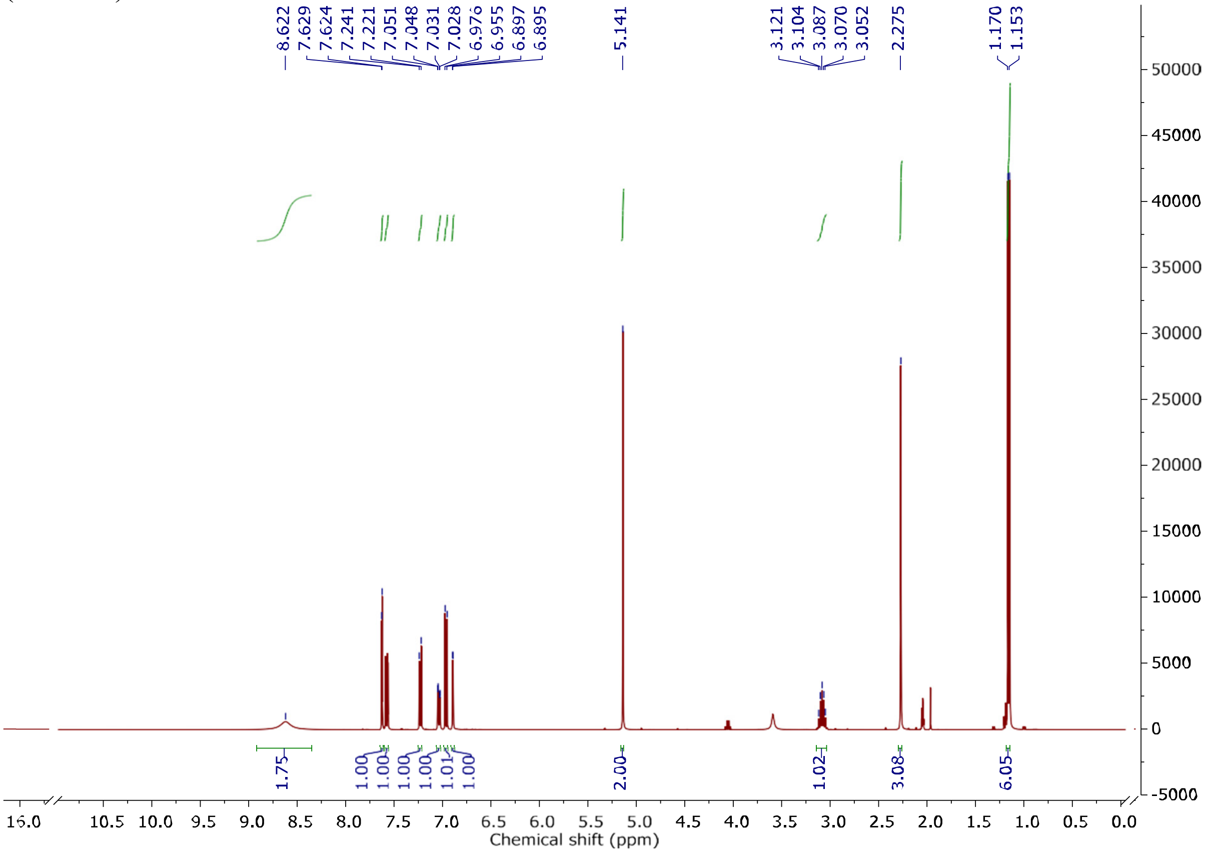

C-NMR

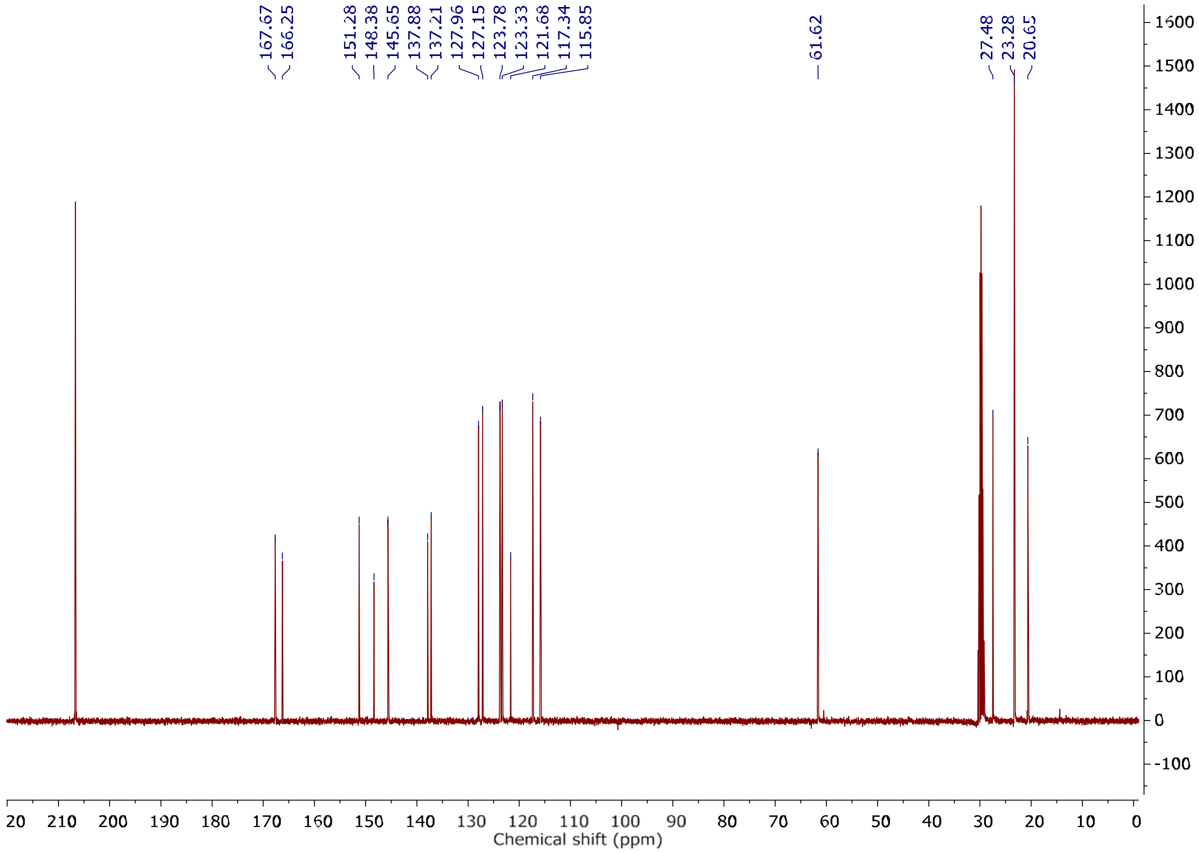
167.67
166.25
151.28
148.38
145.65
137.88
137.21
127.96
127.15
123.78
123.33
121.68
117.34
115.85
61.62
27.48
23.28
20.65
Chemical shift (ppm)

Compound **3** (GAT)
H-NMR

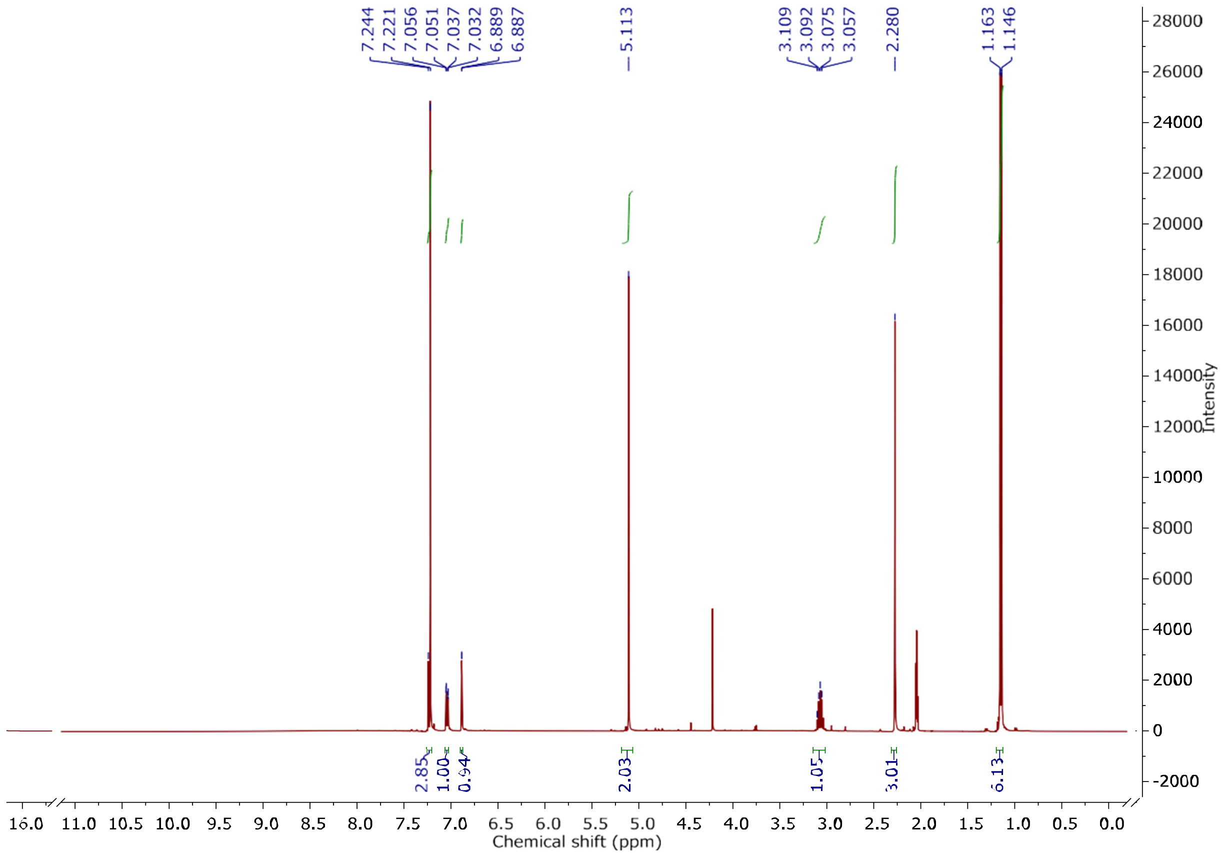

C-NMR

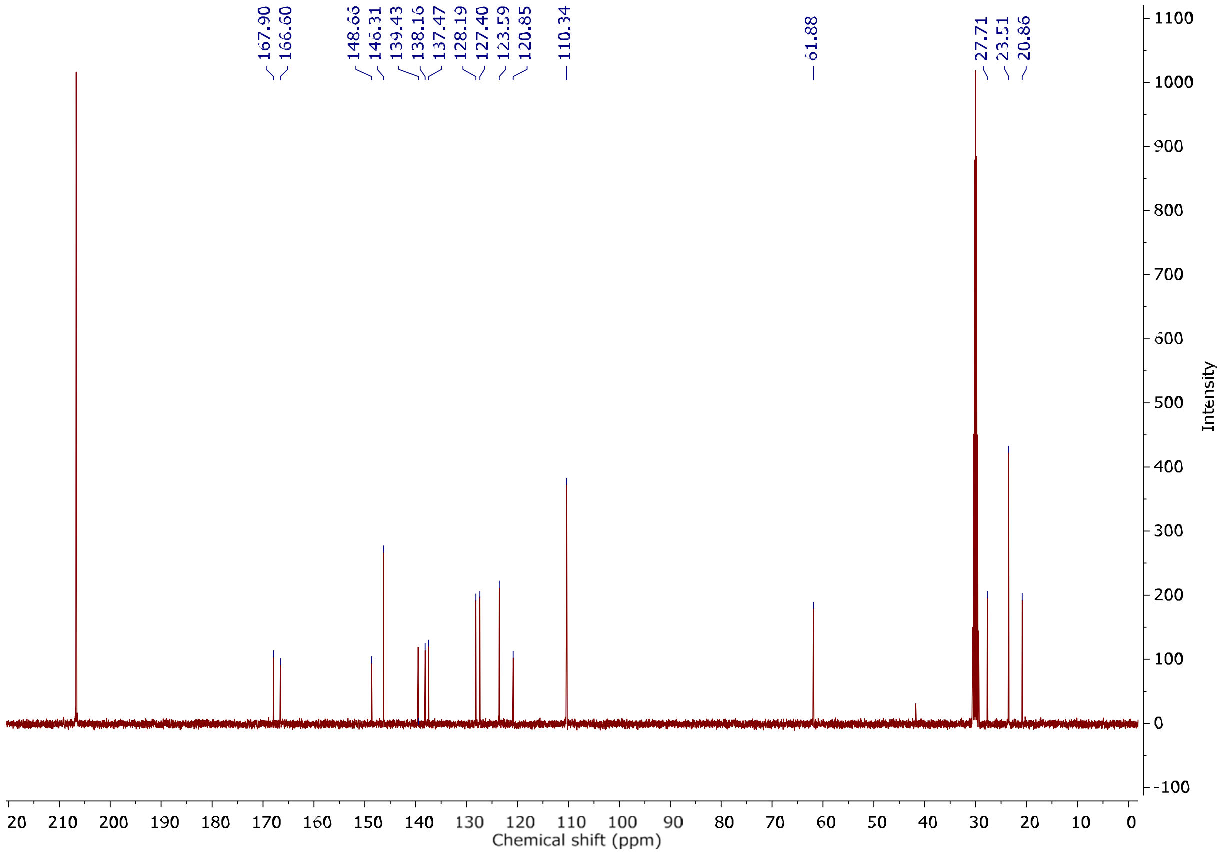

167.90
166.60
148.66
146.31
139.43
138.16
137.47
128.19
127.40
123.59
120.85
110.34
61.88
27.71
23.51
20.86
Intensity
Chemical shift (ppm)

Compound **4** (CT)
(H-NMR)

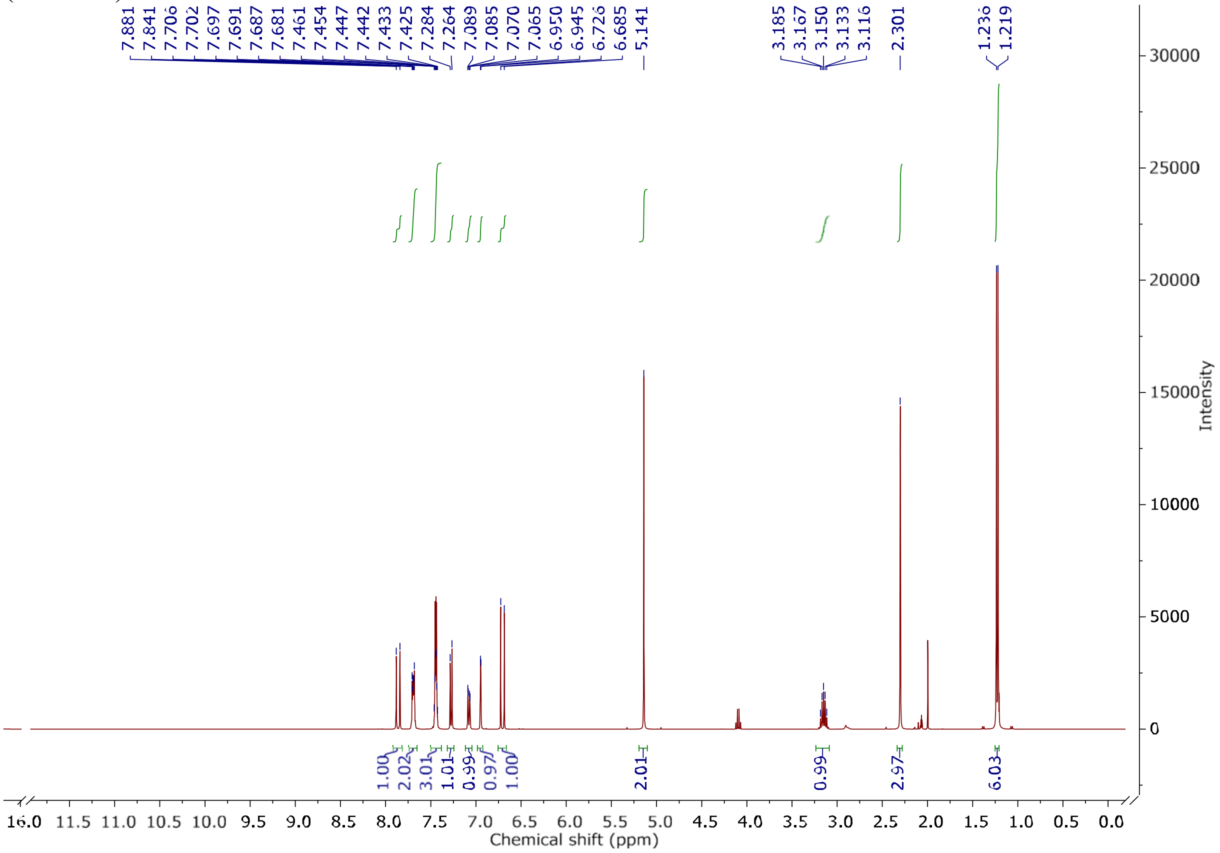

C-NMR

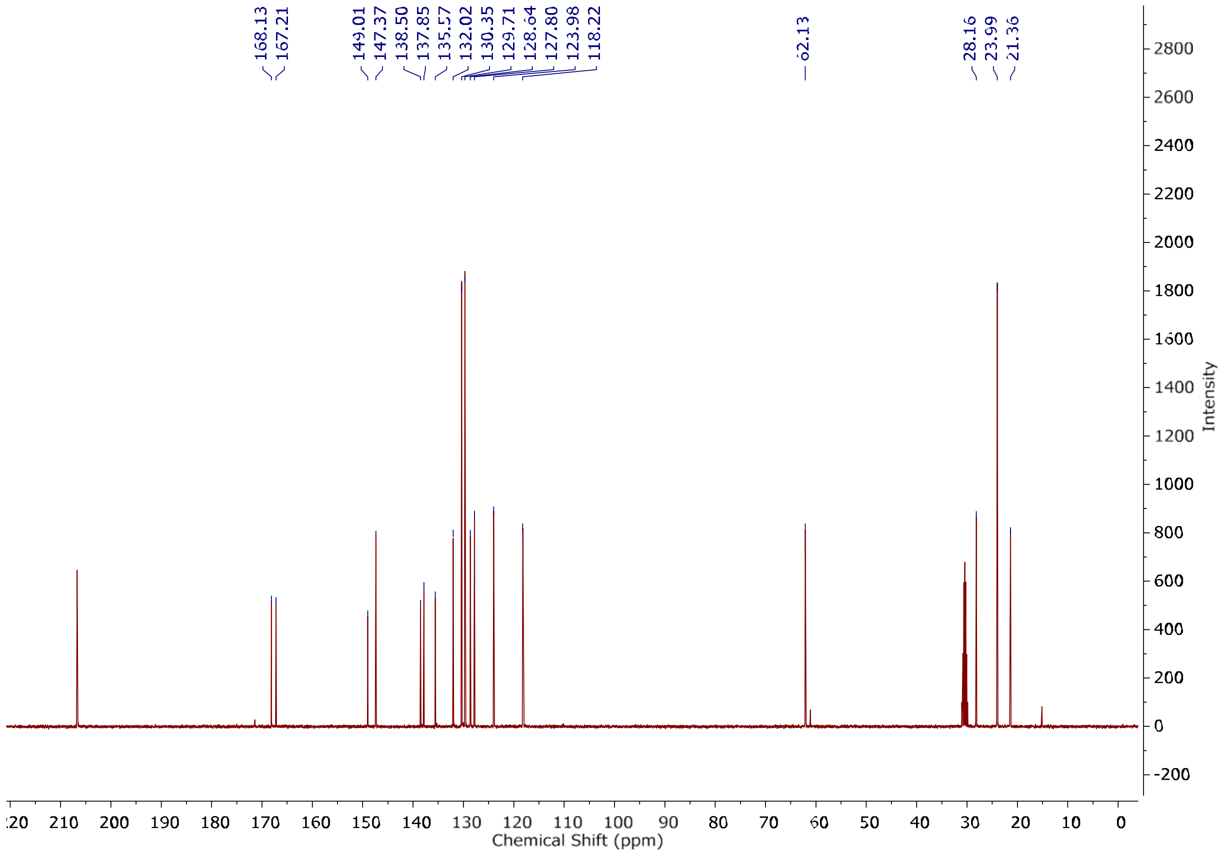
168.13
167.21
149.01
147.37
138.50
137.85
135.57
132.02
130.35
129.71
128.64
127.80
123.98
118.22
62.13
28.16
23.99
21.36
Chemical Shift (ppm)
Intensity

Compound **5** (4-OHCT)
(H-NMR)

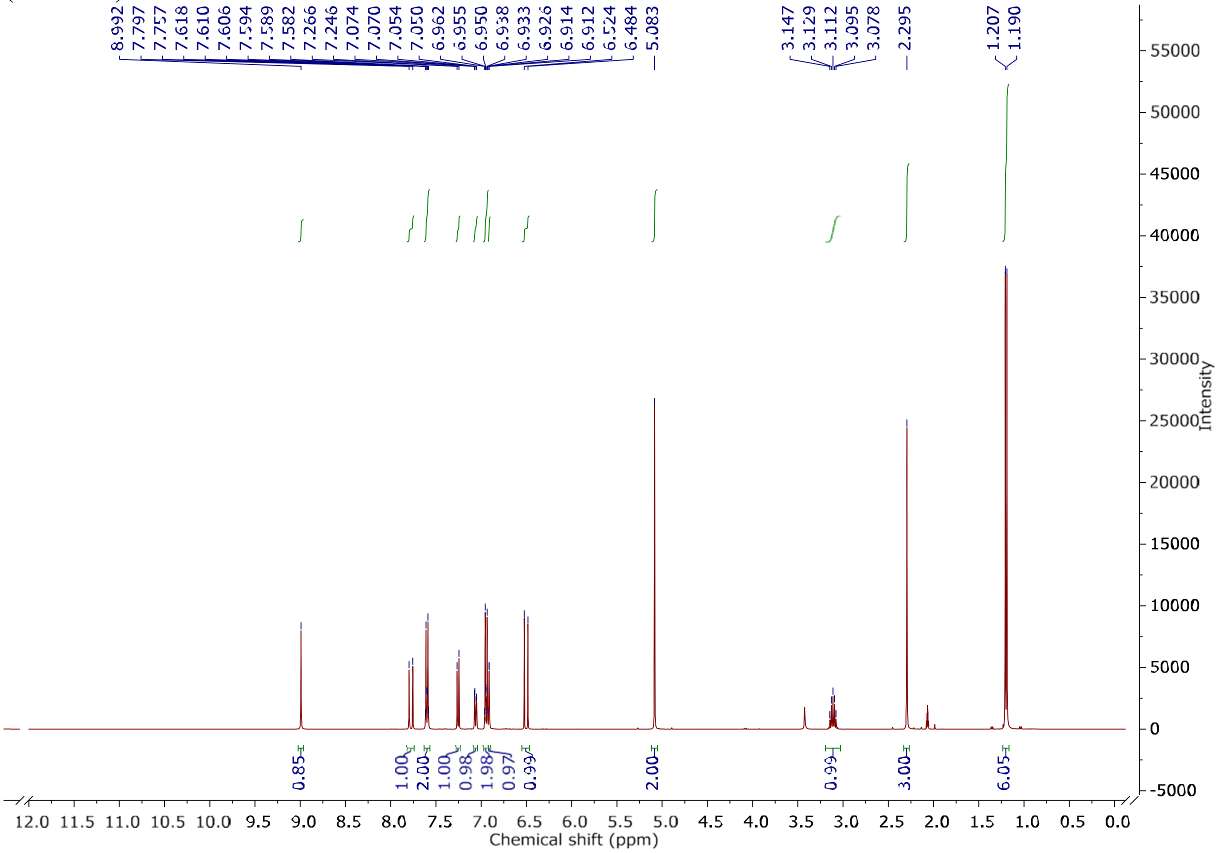

C-NMR

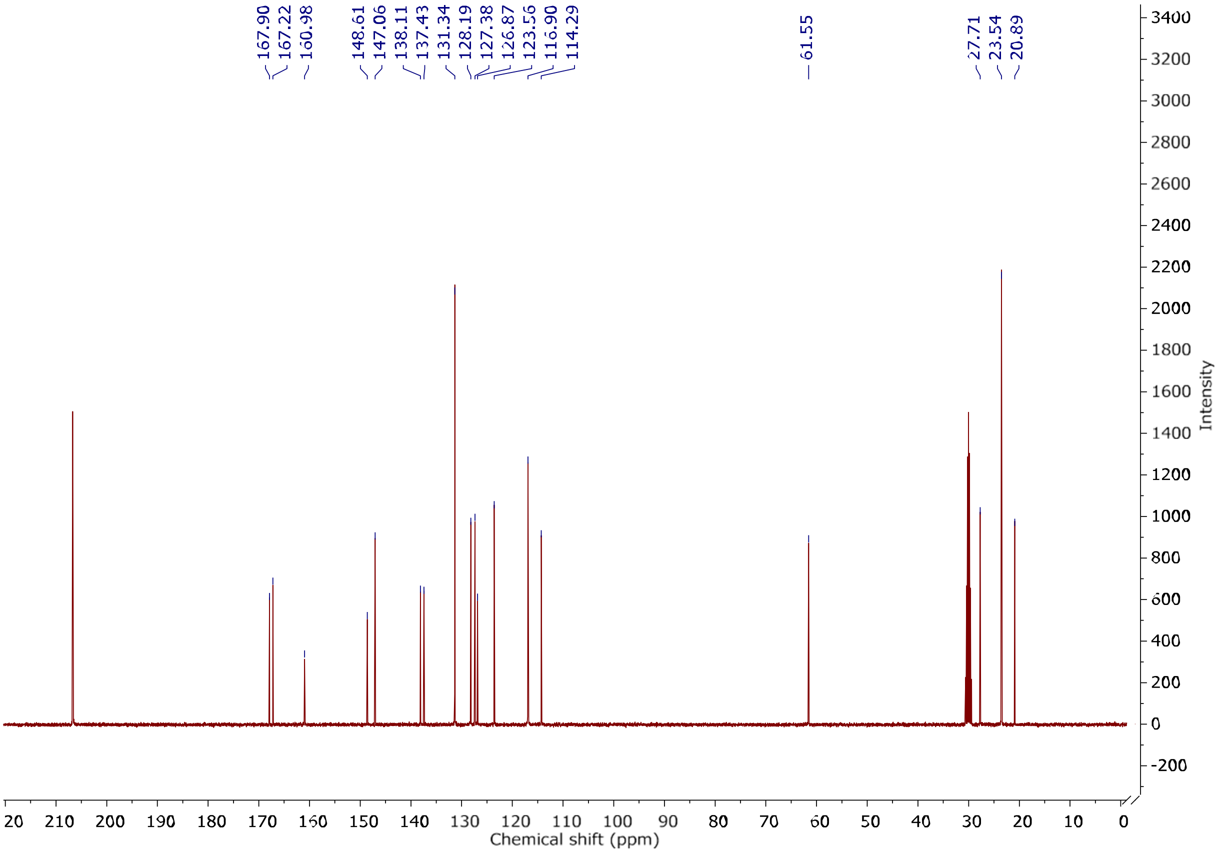

167.90
167.22
160.98
148.61
147.06
138.11
137.43
131.34
128.19
127.38
126.87
123.56
116.90
114.29
61.55
27.71
23.54
20.89
3400
3200
3000
2800
2600
2400
2200
2000
1800
1600
1400
1200
1000
800
600
400
200
0
-200
Intensity
20
210
200
190
180
170
160
150
140
130
120
110
100
90
80
70
60
50
40
30
20
10
0
Chemical shift (ppm)

Compound **6** (CCT)
H-NMR

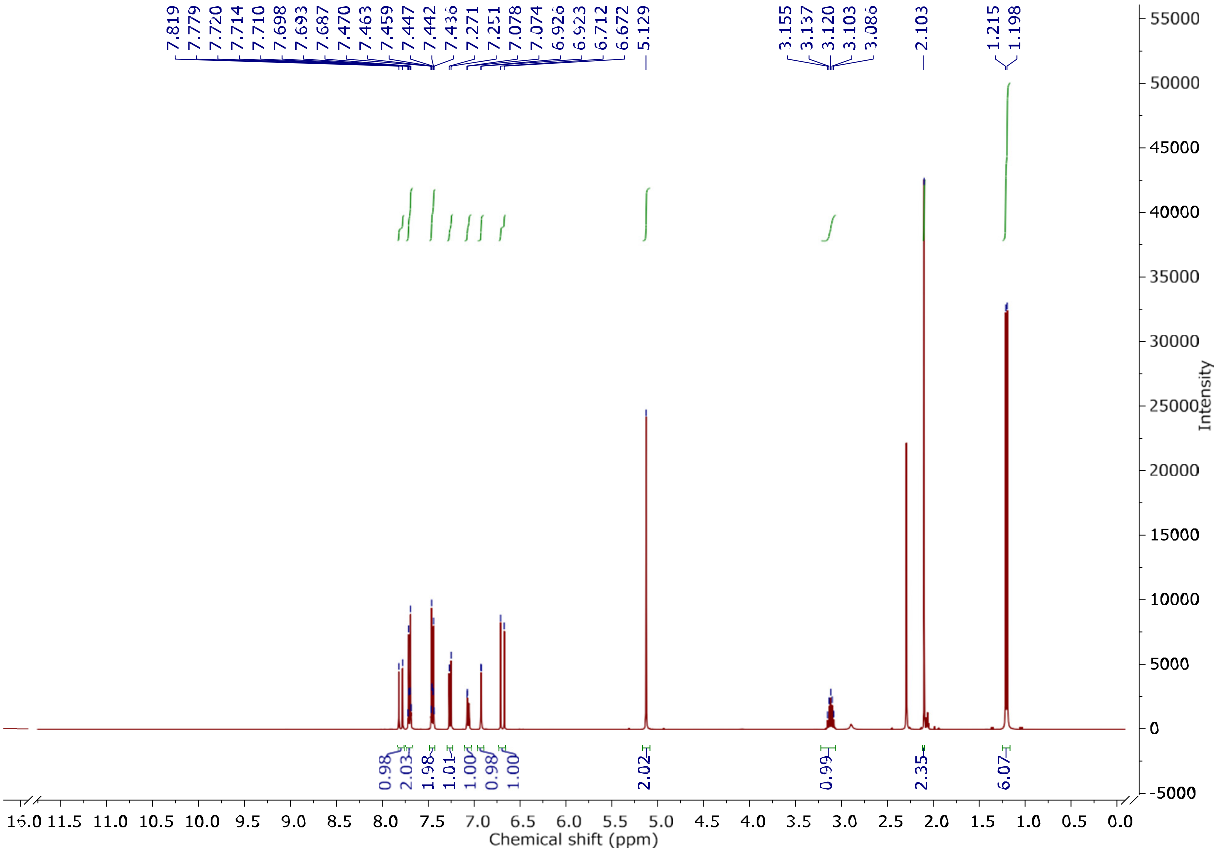

C-NMR

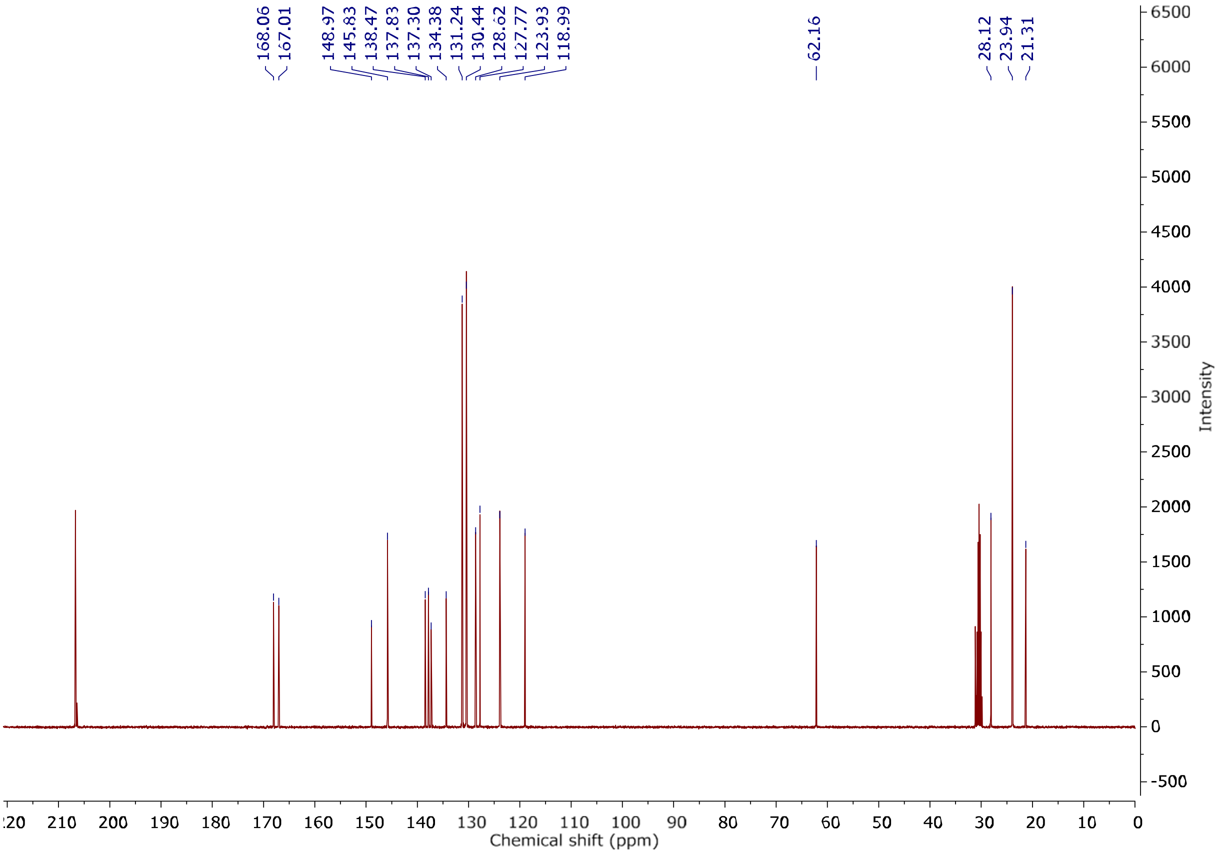

168.06
167.01
148.97
145.83
138.47
137.83
137.30
134.38
131.24
130.44
128.62
127.77
123.93
118.99
62.16
28.12
23.94
21.31
Intensity
Chemical shift (ppm)

Compound **7 (2,4-OHCT)**
H-NMR

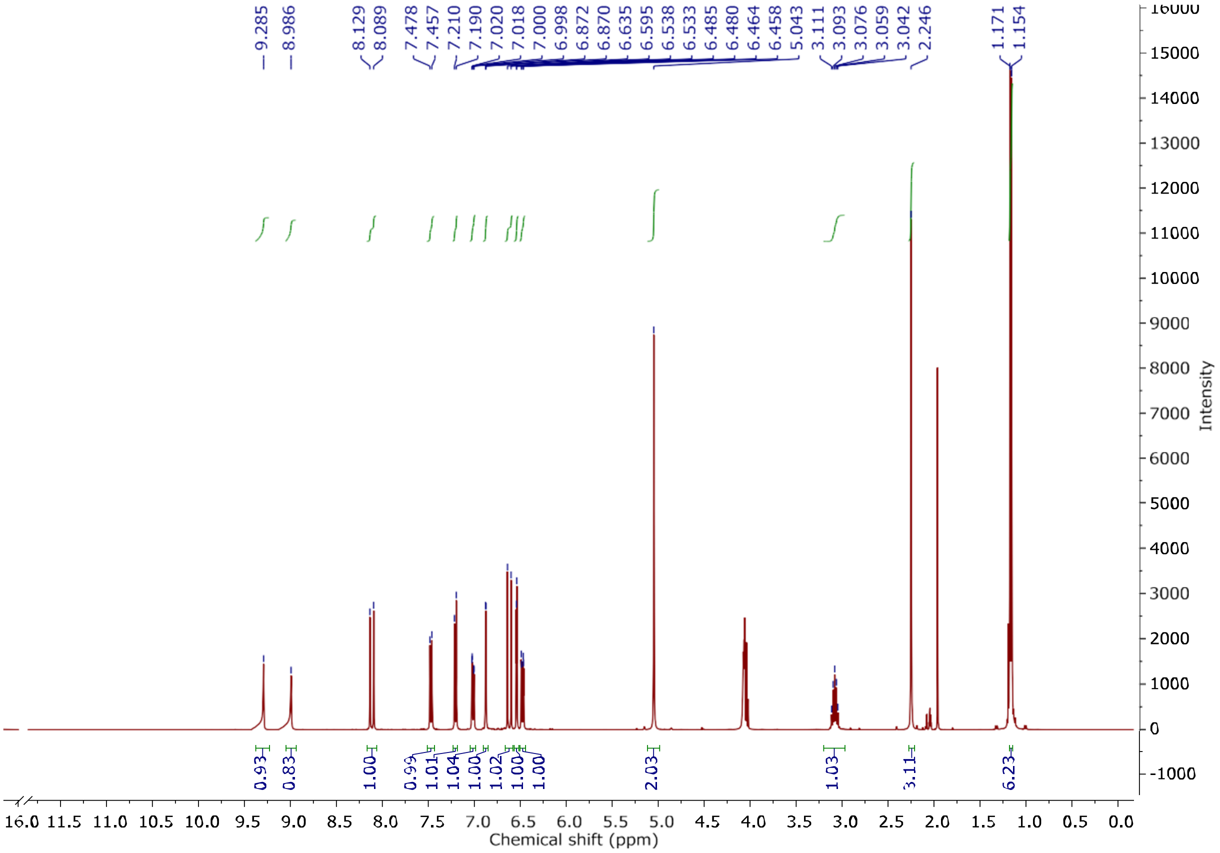

CNMR

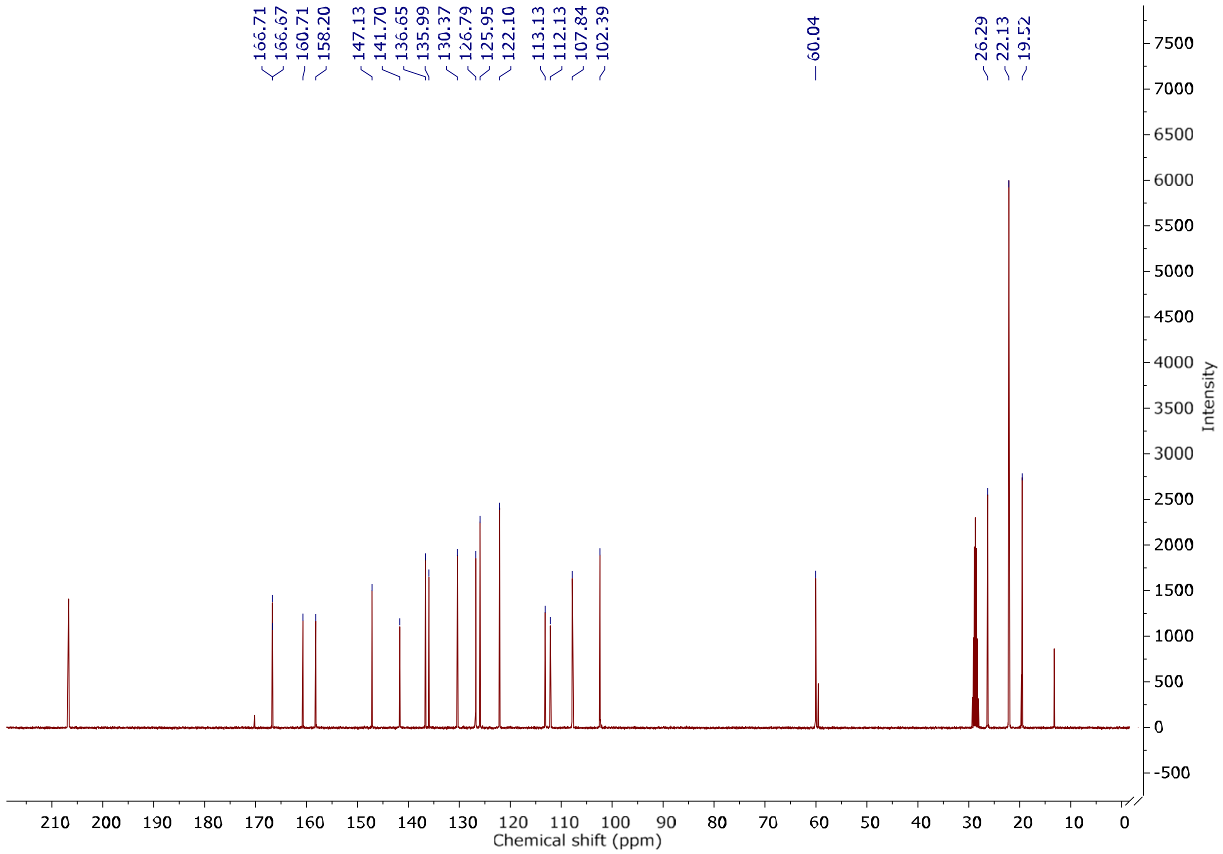
166.71
166.67
160.71
158.20
147.13
141.70
136.65
135.99
130.37
126.79
125.95
122.10
113.13
112.13
107.84
102.39
60.04
26.29
22.13
19.52
Intensity
Chemical shift (ppm)
